# Enhancing brand experience and brand authenticity: The role of octomodal mental imagery and social presence

**DOI:** 10.1371/journal.pone.0321883

**Published:** 2025-04-29

**Authors:** Min Li, Wonjun Chung

**Affiliations:** 1 Department of Design, Tongmyong University, Busan, Republic of Korea; 2 School of Art and Design, Nanjing Audit University Jinshen College, Nanjing, China; Beijing University of Technology, CHINA

## Abstract

This study investigated the impact of Octomodal Mental Imagery (OMI) on brand experience and authenticity in advocating sustainable development and responding to the lack of brand experience and customers’ growing demand for authentic brands. The research employed online questionnaire surveys and data collection via Sojump, resulting in 428 valid responses. The collected data were subjected to quantitative analysis, and the study’s hypotheses were tested using structural equation modeling. The results showed that all the sensory attributes of OMI (visual, auditory, tactile, gustatory, olfactory) positively influenced customers’ brand experience. All the structural attributes of OMI (autonomy, spatial, kinesthetic) positively influenced customers’ brand experience. This study also found that customers’ brand experience positively influenced brand authenticity, while social presence positively moderated the relationship. This study provides branding managers and scholars with a new reference point and scientific data support for companies to implement brand strategies and marketing models, which will help brands to maintain sustainable development in a competitive business environment.

## 1. Introduction

With the rise and continued evolution of online reviews and social media platforms, consumers can now communicate with brands in real time [1]. Businesses are equally quick to obtain feedback and suggestions from users (current or potential consumers) [2]. As a result, today’s customers are confronted with the ever-expanding commercialization of services and products and the globalization of markets [3]. Customer experience has the potential for great diversity as customers may pursue various services or products, and each stage of the consumer journey (search, purchase, use, and post-sale stages) involves different kinds of experiences [4,5]. Business managers also recognize that differentiation requires more than great features and functionality and that how a brand delivers its product is more important than what it offers. In an era of cuts to marketing budgets, businesses will be more focused on expanding experiential marketing to deliver a better service [6]. However, economic value is no longer limited to providing high-quality services but also encompasses a unique brand experience [7]. The importance of this trait continues to rise as consumers are increasingly focused on brand authenticity during their experiences [1,8]. Customers’ growing desire for brand experiences highlights the critical importance of brand authenticity in fostering positive customer interactions [9]. With the popularity of online shopping and the diversification of consumer experiences, enhancing the brand shopping experience has become a crucial topic [10]. Brands must continuously innovate and integrate new technologies to meet consumer needs in order to stay relevant [11]. This is why an increasing number of brand managers are shifting their focus from products and services to the customer experience [12,13]. Therefore, in the current competitive business environment, enhancing the brand experience and ensuring brand authenticity are essential to building a solid relationship between consumers and brands, promoting positive purchase intentions [14], and developing a more comprehensive customer experience model that considers factors that contribute to the overall brand experience, all with the intention of achieving sustainable business development [7].

The study of brand experience has gained increasing attention with the intensification of market competition and the increase in consumer engagement. Many scholars have studied the concept of brand experience from different environments and perspectives, which has led to a more comprehensive and in-depth understanding of this phenomenon [15]. Holbrook and Hirschman [16] first proposed the concept of experience in consumer perception, which opened up the exploration of experience research. Subsequently, Schmitt [17] expanded upon this theory and introduced the concept of brand experience, highlighting that it encompasses a series of sensory, emotional, cognitive, and behavioral responses that consumers exhibit when interacting with a brand. These responses can be triggered through direct (e.g., product use and service experience) or indirect interactions (e.g., advertising word-of-mouth, etc.) [18]. He conceptualized brand experience as a subjective customer response triggered by the experiential qualities associated with a particular brand. Subsequently, Brakus et al. [18] further expanded this conceptualization by emphasizing the multidimensional nature of brand experience and pointing out its comprehensive impact on consumers. In addition, researchers have explored the drivers and influences of brand experience. For example, Chen et al. [19] explored the impact of consumer engagement behavior on brand experience from the perspective of customer motivation, revealing the importance of motivation in brand experience formation. This suggests that consumers’ brand experience is not only the result of passively accepting brand stimuli but is also closely related to their active participation. In the context of the experience economy, the construction of brand experience is increasingly emphasized by enterprises because it enhances brand value and strengthens the emotional connection between consumers and brands [20]. In the internet era, the rapid expansion of social networks greatly boosts the interactivity and dynamism of brand experiences, creating new opportunities for engagement. For example, Morgan-Thomas and Veloutsou [21] proposed the concept of dynamic brand experience, pointing out that brand experience not only occurs in the consumption stage but also throughout the interaction between the brand and stakeholders in the production and consumption process, reflecting the continuity and interactivity of brand experience. Wongkitrungrueng and Suprawan [20] explored brand experience in the virtual environment from a metaverse perspective, emphasizing the value of multidimensional, interrelated experiences and their profound impact on consumer brand perception and behavioral responses. With the rise of social networks and the integration of virtual environments, brand experience includes not only traditional sensory experiences such as visual, auditory, tactile, gustatory, and olfactory but also structural attributes such as spatial, kinesthetic, and autonomy [22]. This indicates that the development of brand experience is shifting towards a more comprehensive and immersive approach to address the evolving needs of modern consumers for brand interaction.

Many scholars have worked on studying consumer preferences for authentic branded products to enhance their brand experience [23]. At present, consumers pay more attention to brand authenticity as a more critical purchasing criterion than quality [24]. Modern consumers aspire to experience authenticity in all aspects, and their constant quest for authentic brand experiences reveals the essential role of brand authenticity in developing loyal consumers [9]. As a result, brand authenticity has gradually become a topic of focus in brand management and marketing research in recent years [25]. Brand authenticity is commonly defined as the alignment between a brand’s behaviors, promises, and core values, reflecting its ability to reliably fulfill its commitments and preserve its essence across various contexts [26]. Much of the existing literature focuses on the relationship between brand authenticity, loyalty, and trust [1] [27]. These studies suggest that when consumers perceive brand authenticity, they are more likely to establish an emotional connection with the brand, enhancing their brand loyalty [14]. However, most existing studies focus on a single cultural or market context and need a comprehensive exploration of brand authenticity in different cultural contexts. This single perspective may lead to limitations in the generalizability of the research results. In addition, most existing studies focus on brand loyalty and brand trust as the primary outcome variables, with less attention paid to the relationship between brand authenticity and other potential variables [27]. However, in the existing literature, brand authenticity is widely regarded as an essential factor for brand differentiation [28]. That said, brand managers still face the challenge of adapting to market changes while enhancing brand authenticity and upholding core brand values. This challenge is especially pronounced in the fast-evolving digital market environment. [26].

Safeer et al. [27] examined the impact of brand experience on brand authenticity and brand liking from the perspective of multidimensional brand experience. However, their research methodology was relatively homogenous and generic. The most recent study by Park et al. [14] also examines the impact on brand authenticity from the perspective of multidimensional brand experience but with the addition of moderating variables and a focus on retail business models. On the other hand, Khan et al.’s study [3] examines brand experience and authenticity through the moderating variables of individualism and collectivism in tourism. Hsu [29] focuses on brand experience, liking, and repurchase intent from a gamification perspective. In addition, some studies examine the relationship between brand experience and attachment [30], brand strength [31], brand equity [32], and brand loyalty [33]. Although existing studies have made some progress in brand experience and brand authenticity, the complexity and diversity of the relationship between the two require a more comprehensive and nuanced analytical framework. To this end, the Octomodal Mental Imagery (OMI), a comprehensive imagery measurement tool that provides both long and short versions for a variety of contexts, covers sensory (i.e., visual, auditory, tactile, gustatory, and olfactory) and structural attributes (i.e., autonomy, spatial, and kinesthetic). Belonging to the second-order formative constructs, the OMI provides a multidimensional perspective for in-depth analysis of the relationship between brand experience and brand authenticity [22]. These eight sensory or cognitive channels make it possible to describe and understand the internal mental imagery or experiences formed by an individual in a specific context. As an emerging theoretical framework, OMI emphasizes multisensory and structural dimensions of mental experience and explores how these mental stimuli combine to influence an individual’s cognitive, affective, and behavioral responses [22].

Over the years, mental imagery has been widely used as an influence strategy in marketing [10]. For example, Apple’s slogan “Imagine the Possibilities” and Samsung’s “Imagine” aim to stimulate consumers’ imagination and significantly influence their evaluations and behaviors [10]. In travel and tourism marketing research, mental imagery is acknowledged as a crucial cognitive process during the pre-and post-travel phases. It has been extensively studied in related areas [34]. Imagery directly affects consumers’ expectations and their recall of the post-travel experience. Consumers can recall previous travel experiences through vivid imagery, enhancing the feeling of recollection [35]. The tourism-related literature also suggests that imagery plays a crucial role in travel consumption [36], making OMI an essential tool for assessing the sensory and creative elements of enhanced hospitality and travel experiences [37]. However, there has been a relative lack of research on OMI since it was developed and validated by Khalilzadeh et al. in 2023 [22]. Only Zhu and Chung [38] have investigated the impact of OMI on shopper experience and mall loyalty from the perspective of shopping centers, and their study showed that all attributes in OMI except auditory, autonomy, and kinesthetic positively affect shopper experience. There is currently a lack of research investigating how brand experience and authenticity can be enhanced from a more comprehensive synthesis of OMI [22]. Through eight key dimensions, i.e., visual, auditory, tactile, gustatory, olfactory, autonomy, spatial, and kinesthetic, OMI can provide insights into how consumers form brand perceptions through different sensory and structural attributes during their engagement with a brand, further influencing their perception of brand authenticity. Therefore, the selection of OMI as a research variable aims to provide a multidimensional perspective that helps us to fully understand all aspects of brand experience, thereby providing deeper insights into the relationship between brand experience and brand authenticity.

This study fills this theoretical gap by systematically exploring the effects of eight mental imagery attributes—visual, auditory, tactile, gustatory, olfactory, autonomy, spatial, and kinesthetic—on brand experience and brand authenticity by applying the OMI scale. OMI provides a detailed framework for revealing how consumers form an overall impression of a brand under multisensory stimuli. It also considers structural attributes, which are crucial for understanding brand authenticity and its role in brand perception. Moreover, Khalilzadeh et al. [22] confirmed that all conditioning and imagery processes appear in the three structural attributes (autonomy, spatial, and kinesthetic). By integrating OMI into brand research, this study constructs a multilevel theoretical framework that reveals how brand experience affects the formation of brand authenticity under the combined effect of multisensory and structural attributes. The introduction of OMI fills a gap in the existing literature. It provides a new strategic perspective for brand management and a practical guide to designing experiences with multisensory and structural aspects in brand marketing practices.

Meanwhile, social presence, an essential concept in e-commerce and online interaction, has recently attracted a great deal of attention. It is defined as the salience of others in mediated communication and the resulting salience of interpersonal interactions [39]. It shows how strongly individuals perceive the presence of others in digital or virtual spaces. Although existing studies have explored the impact of social presence on consumer behavior and decision-making, such as in e-commerce environments, where social presence has been shown to influence consumers’ impulse buying behavior [40] and enhance their purchase intentions [41], its application in brand experience and brand authenticity studies is still lacking. First, although studies have identified the critical role of social presence in digital platforms, they have mainly focused on its direct effects on consumer behavior, such as impulse buying and trust enhancement [40,41]. However, there is a relative dearth of research on how social presence moderates the relationship between brand experience and authenticity [14]. This suggests that the role of social presence in the complex psychological mechanisms of brand experience has yet to be fully explored, and this research gap provides an important research direction for this study. Second, the current literature mainly focuses on the influence of social presence at the individual level and less on its role at the group or societal level [42]. This limitation may lead to a lack of generalizability of the findings in complex real-world situations. Therefore, social presence is introduced into the OMI theoretical framework in this study to understand in more depth the role of social presence in brand experience and its impact on brand authenticity. Finally, although some studies have explored the role of social presence in consumer–brand interactions, such as instantaneous communication through social networks that enhances engagement in brand experience [43], existing studies still do not fully reveal the role played by social presence in the formation of brand authenticity. How social presence, as an essential perceptual element [44], affects brand authenticity in different cultural contexts and market environments remains an area for in-depth research. Meanwhile the moderating variable of social presence is added in this study to make the analysis more comprehensive. This extends the existing brand marketing literature, verifies the generalizability of the theoretical model, provides references and information for other subsequent studies, and helps deepen the understanding of brand interaction in the digital era. In addition, it provides a more precise roadmap for business executives and brand managers to implement brand experience as a strategy to enhance brand authenticity from the OMI dimensions. This study also demonstrates the motivation and inspiration of the researchers to conduct this study from the above perspectives.

Existing studies do show that brand experience has a significant effect on brand authenticity [27]. However, research is still lacking on the relationship between brand experience and brand authenticity, particularly when moderated by social presence. Many existing studies have focused on the intrinsic dimensions of brand experience [45–47] and specific aspects of brand authenticity [1,8,26], with a singular framing and theoretical perspective that neglects the potential impact of social presence as a moderating variable. In addition, we identify several critical gaps and inconsistencies in the existing literature. First, although some studies have explored the direct impact of brand experience on brand authenticity, the role of social presence in this context requires more attention [27]. Second, existing studies have mainly focused on the intrinsic dimensions of brand experience or limited contexts, necessitating a comprehensive analysis of the relationship between mental imagery on brand experience and brand authenticity [3,14]. Third, existing empirical studies have methodological limitations, and thus cannot fully describe how social presence moderates the relationship between brand experience and brand authenticity [29]. Therefore, based on past research and in order to fill these gaps and lacunae, this study attempts to comprehensively construct a theoretical model of brand experience and brand authenticity from the perspective of OMI, combined with social presence as a moderating variable, aiming to comprehensively explore how brands strengthen perceptions of brand authenticity by influencing consumers’ affective responses and cognitive processes. Brand experience is critical to the formation of brand perception and the core foundation of brand authenticity. OMI provides a unique perspective to analyze this complex process by integrating sensory and structural attributes, revealing how brands can enhance consumers’ perceptions of brand authenticity through well-designed multidimensional experiences. Social presence as a moderating variable further enhances the impact of brand experience on brand authenticity, especially those that combine sensory and structural attributes, making the perception of brand authenticity deeper and more enduring by increasing consumer trust and social interaction. By integrating brand experience, authenticity, OMI, and social presence, this study constructs a comprehensive framework that reveals how multidimensional experiences and social environments synergistically enhance brand authenticity, providing theoretical support and practical guidance for brands to establish an advantage in a competitive market.

In addition, the number of studies in the literature on brand experience in Western countries significantly exceeds that in Eastern countries, and the behavioral differences between Eastern and Western consumers underline the need for such studies in China [7]. The choice of China for this study is based on the following unique socio-cultural and economic characteristics: First, China’s unique socio-cultural context is heavily influenced by Confucianism and emphasizes collectivism, harmonious relationships, and the culture of saving face [48]. In this context, the brand is a symbol of the product and a reflection of social status and personal identity [49]. Therefore, brand experience has a unique cultural expression in China, which provides a different perspective from that of the Western market and helps to reveal the complex relationship between brand experience and brand authenticity in a different cultural context. Second, as the second largest economy in the world, China has a sizeable middle class [50], and the dynamics of economic development have caused consumers to gradually shift from pragmatic consumption to sensual consumption that emphasizes brand experience and value [51]. At the same time, consumers’ increasing demand for brand authenticity [1,8] provides an essential real-world context for studying how brand experience affects brand authenticity. Finally, China has a high level of digital and social media penetration and a booming digital economy, and social media platforms play an essential role in consumers’ lives, creating a highly digitalized and socialized brand experience environment [52]. In this context, how brands can enhance brand experience through mental imagery and then influence brand authenticity is a forward-looking research topic, and this market environment provides a rich practical scenario for applying OMI theory.

This study aims to validate the effects of OMI on brand experience and brand authenticity through structural equation modeling (SEM). The objectives are to clarify how individual sensory and structural attributes in OMI affect brand authenticity through brand experience and to explore the role of social presence, a moderating variable, in this context. The core hypothesis of this study is that these mental imagery attributes can significantly enhance brand experience and further improve brand authenticity, thus not only enriching the theoretical research on brand experience and brand authenticity but also providing empirical support for brand management practices. This study adopts a quantitative research design, aiming to systematically reveal the comprehensive impact of each sensory and structural attribute of OMI on brand experience. The data were gathered using an online questionnaire. We utilized the Questionnaire Star platform to distribute the questionnaire and collected 428 valid data points. The questionnaire was designed to address existing OMI scales and combined with relevant theories of brand experience and brand authenticity to ensure the content of the questionnaire had scientific rigor. Participants were chosen using a combination of simple random and purposeful sampling techniques to guarantee the data’s representativeness and validity. The collected data were quantitatively analyzed using SPSS25.0 and AMOS23.0 software, and were initially analyzed statistically to assess their internal consistency and validity. Subsequently, the research hypotheses were tested using SEM to explore the relationship between the variables and assess the model’s fit. This comprehensive analysis method can be used to effectively validate the structure and path relationships of the theoretical model, thus ensuring the accuracy and scientific validity of the research results.

This study begins by reviewing OMI, brand experience and authenticity, and social presence to understand which factors influence brand experience and authenticity and the relationship between the two, formulate the appropriate hypotheses, and develop a theoretical model. In Section 3: Materials and methods, the measurement scales are developed, and the research methodology is described. In Section 4: Results, the findings of the quantitative analysis are derived. In Section 5: Discussion and implications, the findings are discussed and the theoretical and practical implications, as well as the limitations, are presented. The article concludes with a full summary and describes the directions of this study for future research.

## 2. Literature review and hypotheses

This study is based on two critical theoretical constructs: the OMI [22] and brand experience [18]. First, the OMI scale provides a theoretical framework comprising five sensory attributes (visual, auditory, tactile, gustatory, and olfactory) and three structural attributes (autonomy, spatial, and kinesthetic). The OMI theory encompasses psychological processes related to perception, cognition, and emotion, emphasizing how individuals’ perception and experience of a brand influence their attitudes and behaviors toward it, which applies to experiential consumption. Second, brand experience theory focuses on the holistic perception and emotional experience generated by individuals’ interactions with a brand, emphasizing how brand marketing activities shape consumers’ overall perception of the brand. The two theoretical frameworks are intertwined, and together they build the study’s theoretical foundation. By integrating these two theories, this study aims to enhance comprehension regarding the connection between brand experience and authenticity. This underlying theoretical framework provides an integrated, multi-layered perspective to reveal how sensory and structural attributes in OMI shape brand experience.

### 2.1. Octomodal mental imagery (OMI)

Neuropsychology defines mental imagery as a quasi-perceptual experience that arises without an actual stimulus and is expressed as a sensory image-like representation in the human brain [53]. Mental imagery is a third-order construct with sensory and structural formative properties [22]. While a layperson may limit imagery to the visual, scholars who study mental imagery take a broader view, extending the concept to the imagery of the entire bodily experience [54]. Schifferstein [55] conceptualized mental imagery as internally generated representations of objects, scenes, or events that evoke vivid sensory images in all sensory modalities. It is a concept that encompasses multiple dimensions [56,57] and is self-generated according to subjective mental processes [58]. In 2022, Elder and Krishna refined their definition of mental imagery, stating, “Mental imagery is a prospective, multimodal sensory and cognitive representation formed through the automatic or intentional recall of memory” [10]. Huh et al. [59] also noted that consumers often use mental imagery to facilitate decision-making. In hedonic consumption, imagination visualizes past experiences that have already occurred, while anticipation is the ability to experience the future in advance by mentally simulating events or objects that have not yet occurred [60]. Imaginative brand experiences allow consumers to interact with brands retrospectively and prospectively [10]. Thus, mental imagery is a critical concept in consumer psychology that represents perceptual representations in memory and is often seen in marketing research as an essential mechanism for marketing stimulus processing [61].

Khalilzadeh et al. [22] show that OMI, a comprehensive imagination scale developed based on the neuroscience literature, is the most comprehensive measurement tool developed in the imagination literature and is beneficial in experiential consumption. Furthermore, they suggest that OMI can be utilized to evaluate the effectiveness of efforts to enhance the sensory and fantasy aspects of the overall experience. In addition, OMI and the Experience Economy model can significantly enhance the measures they co-create. Elder and Krishna [62] found that rich mental imagery can motivate consumers to create a deeper emotional connection during brand interactions, and this emotional connection not only enhances the pleasure of the brand experience but also strengthens consumers’ identification and loyalty. In addition, mental imagery can stimulate consumers’ imagination through vivid brand stories, advertisements, or marketing campaigns, thus enhancing a brand’s emotional appeal and interactivity [63]. Spence and Fiszman [64] found that brand trust and loyalty are enhanced when consumers perceive the authenticity of a brand through mental imagery. According to Thomas’ study [65], mental imagery is crucial in memory and motivation because it comprises the reproduction of actual perceptions of the past and the anticipation of expectations or fears about future experiences. Elder and Krishna [10] found that imaginative brand experiences inspire consumers to review and have future expectations of a brand. Consumers perceive more robust sensory or hedonic qualities through mental imagery that anticipates the experience of using a product [10]. For example, promotions with stronger product associations, such as “buy a shirt and get matching pants free,” are more effective at eliciting consumer imagery that encompasses sensory and structural experiences and further influences consumer attitudes toward the product compared to simple discount offers [66]. Similarly, vanity sizing drives purchase decisions using smaller labels that evoke associations of a more positive self-image, leading to higher satisfaction in the shopping process [67]. In addition, the mental imagery created when navigating a website affects consumer satisfaction and attitudes toward the brand [68]. Mental imagery has therefore been used as a strategy in brand marketing for many years, and it has the potential to influence consumer judgment and behavior [10,69].

Based on the results of previous research, there is a solid foundation to propose that mental imagery can influence brand experiences and evoke emotional responses in consumers [22]. OMI (released online in early 2023) is a reliable and valid scale, developed and validated through three rigorous studies and six data collections, for measuring imagination and outlook in all aspects of the consumer environment and beyond. It comprises five reflexive components related to sensory properties (visual, tactile, auditory, gustatory, and olfactory) and three related to structural properties (autonomic, spatial, and kinesthetic) [22].

### 2.2. Visual and brand experience

The visual is one of the sensory attributes of OMI, and it plays a crucial role in the brand experience. Humans are visual animals, and visual mental imagery research predominates. Visual perception is vivid and provides vast detailed information, so visual stimuli are often regarded as an essential information transfer tool [70]. It can create a profound consumer interaction through various brand designs, packaging, advertising, and environmental displays [17]. Studies have shown that visual attributes can not only attract consumers’ attention quickly but can also convey the core message of a brand through colors, shapes, and images, thus shaping how consumers perceive its authenticity and their overall experience with it [71]. They hold significant importance in the cognitive, emotional, and behavioral dimensions of branding, and past research has focused extensively on this issue [46].

Visual form is one of the main tools in brand environments, and its importance lies in its ability to process input from multiple sources simultaneously, thus providing a coherent representation of the external environment [72]. Sample et al. [73] found that approximately 70% of human cognition originates from visual form. During the customer journey, visual neurophysiological data provided by a brand are collected when the body interacts with a touchpoint; these data are sent through neural circuits to command centers in the brain and ultimately realized as a sensory brand experience [46]. Zha et al.’s study [74] also demonstrated that brand data mined by visual cues in the brand’s environment are processed internally as a sensory brand experience, impacting customer–brand connections regarding essential factors such as customer satisfaction, brand loyalty, and favored customer indicators. In the digital consumer environment, the importance of the visual experience has increased. With the growth of e-commerce and social media, a brand’s online presence is now crucial. When consumers engage with brands through online platforms, visuals become the primary medium of interaction, significantly influencing their purchase decisions and brand experience [75]. For example, Sinha and Fukey [76] found that beautiful visual design in e-commerce platforms enhances consumers’ browsing experience and significantly increases their purchase intention and brand loyalty. Meanwhile, Hagtvedt and Patrick [71] suggested that brands can enhance their cultural connotation and uniqueness through visual art elements, thus improving their authenticity and malleability.

Thomas’ [77] study conceptualized mental imagery as a verbal description of a visual scene. Visual elements such as brand logos, package designs, advertisements, and store layouts can attract consumers’ attention and directly influence brand experience [78]. Li and Lin’s study [79] found that appealing visual designs and displays can enhance brand attractiveness and memorability, increasing consumer perception of the brand and its favorability. Furthermore, Kim and Sullivan [80] found that visual stimuli can also trigger an emotional response, which helps consumers establish an emotional connection and strengthens brand loyalty. Liu et al.’s study [81] demonstrated that visual information on branding conveys a wealth of information about the user’s consumption experience, attitudes, and feelings, which can influence the customer’s behavior towards the brand experience. For example, the color of orange juice can evoke sensory responses that influence brand preference [82]. Brand logo design and color choices directly influence consumers’ affective experiences and emotional responses, affecting their attitudes and loyalty [83]. Visual elements can enhance a brand’s emotional connection by creating aesthetic pleasure and a more profound consumer experience during brand interactions [84]. Bloch et al. [84] further noted that individual differences in visual product aesthetics directly affect consumer attitudes toward the brand and purchase intentions, suggesting that visual aesthetics have a broad and profound impact on brand experience. In addition, the visual appeal of store layouts and display designs can prolong consumers’ in-store stays and attract their attention, thus increasing their purchase intention [85].

Therefore, the visual component, as an essential aspect of OMI, has a multi-dimensional impact on brand experience, covering aspects such as brand recognition, esthetic pleasure, and emotional connection and playing a crucial role in digital and physical scenarios. Not only can it influence consumers’ decision-making process at the time of purchase, but it can also have a long-term positive impact on brand perception and emotional experience. By effectively utilizing visual elements, brands can stand out in a competitive marketplace, enhance consumer loyalty and perception of authenticity, and improve the overall quality of the brand experience. In addition, vision impacts almost all other sensory attributes and some structural attributes (e.g., kinesthetic) [22]. Based on the above theoretical basis, a reasonable hypothesis can be made:

**Hypothesis 1 (H1)** Visual mental imagery has a significant positive impact on brand experience.

### 2.3. Auditory and brand experience

Kosslyn [86] points out that imagery exists in visual, auditory, and kinesthetic forms. The auditory component plays a vital role in brand experience and is a more unique and distant sensory experience than other sensory attributes [87]. Auditory imagery can directly influence the perception of brand distance and can elicit greater customer interest [10]. As the body interacts with touchpoints along the customer journey, it collects the brand’s neurophysiological data (auditory). These are sent through neural circuits to command centers in the brain, where they are processed, evaluated, and transformed into a sensory brand experience [46]. Spangenberg et al. [88] emphasized the concept of environment in their study, where they tested a multi-stimulus model in a Christmas setting to examine the interactive effects of Christmas music and Christmas scents on consumers’ product evaluations and brand experience. Scott et al. [89] found that sound and music choices directly impact a consumer’s emotional state and affective experience, thereby shaping their perceptions and emotions toward the brand. Kim and Sullivan’s study [80] demonstrated that brand marketing campaigns with pleasurable sounds enhance consumers’ emotional engagement and improve their perceptions and trust in the brand. In addition, the consistency and uniqueness of a brand’s voice can help build a more prominent and lasting image in the marketplace. For example, in Intel’s corporate identity rebranding in 2020, the brand logo was changed but the three-second audio logo heard every time an Intel internal computer is turned on was retained. This simple five-note melody became one of the most recognizable elements of Intel’s branding [46]. Spence [90] found that even the sound symbolism of brand names can evoke cross-modal sensations, conveying more subtle meanings about the brand.

The auditory component not only initiates social interaction but also shapes brand emotions and fosters consumer trust through the perception of sound [91]. Li et al. [92] point out that the auditory component also has a supportive role, contributing to synergistic effects between the senses, thus enhancing the likelihood that viewers will take in the message and the brand experience. Krishna and Ahluwalia [93] investigated how language affects young consumers’ responses to advertisements in virtual environments. From a consumer behavioral perspective, adding sound effects to radio advertisements can enhance the vividness of the visual content, thereby triggering a more positive emotional response [92]. Sweeney and Wyber [94] found that soothing background music significantly enhances the shopping experience, making consumers feel more comfortable and relaxed during brand interactions. This, in turn, extends their time spent in-store and increases their purchase intention. Scott [95] also noted that the choice and rhythm of music can enhance the emotional resonance of advertisements, leading to a stronger emotional connection with the brand and increasing its appeal. The flavor and brand images of Coca-Cola are conveyed through a multi-sensory experience. For instance, the auditory features of the “thump” sound from a can of Coca-Cola being opened or the hissing sound of a Coke on ice can evoke the brand image in consumers’ minds and convey the brand’s meaning through internal and external sensory experiences [96]. Auditory elements are increasingly essential in digital and online brand experiences. With the rise of e-commerce platforms and online advertising, brands use sound and music to compensate for the lack of physical touch in online interactions. For example, Doucé et al. [97] found that the choice of background music during online shopping affects consumers’ moods and significantly enhances their shopping experience and brand authenticity.

Auditory stimuli have the explicit function of triggering brand recall and serve as a threat detection tool. Unlike vision, hearing is not limited by the visual span, so it is crucial to create soundscapes that can simulate a brand setting in a non-threatening and relaxed state [74]. As a critical sensory dimension of brand experience, the auditory component can positively impact consumers’ brand experiences through emotional resonance, brand recognition, and enhanced sensory engagement. Based on these points, the following hypothesis can be made:

**Hypothesis 2 (H2)** Auditory mental imagery has a significant positive impact on brand experience.

### 2.4. Tactile and brand experience

The tactile component, one of the most important ways to engage with a brand, requires more physical contact for the whole experience. This hands-on interaction also fosters a psychological closeness between consumers and a brand [87]. Psychological distance also affects the level of detail and color represented in mental imagery [98]. The importance of tactile imagery continues to grow as shopping becomes progressively digitalized, and imagined tactile interactions influence consumers’ perceptions of product ownership and quality [10]. In digital environments, technologies such as virtual and augmented reality have enabled haptic experiences to be partially realized in online shopping and brand presentations despite a lack of actual physical contact for consumers. Haptic simulations in virtual environments can compensate for the lack of haptic experience in online shopping by enhancing consumer immersion, thus enhancing the consumer’s shopping experience and accurate brand perception [99]. Karangi and Lowe [100] found that tactile stimulation enhances consumers’ perceptions and evaluations of a brand’s products or services and triggers their emotional resonance and identification. Leo et al. [101] further found that the degree of pleasure in the tactile experience is closely related to consumer satisfaction and brand loyalty. This finding is particularly significant for high-touch products (e.g., apparel or furniture). Therefore, by designing products that are comfortable to touch and provide a pleasurable tactile experience, brands can effectively enhance consumers’ brand experience and emotional connection.

When the body interacts with contact points during the customer journey, tactile neurophysiological data provided by the brand are collected, transmitted through neural circuits to the brain’s command center, processed, and evaluated to produce a sensory brand experience [46]. Li et al. [92] found that tactility has a supportive role in enhancing the sensory experience and boosting cognitive effects, which helps to increase the viewer’s acceptance of the information and their brand experience. Balconi et al. [102] identified tactility as one of the key senses in providing consumers with a complete and coherent perception of the experience, as it allows for an “enhanced” sensory and cognitive experience through touching the product. This tactile feedback can create a stronger sense of product intimacy and trust through “haptic priming” [103].

Tactile cues establish a direct physical link with customers, offering a tangible aspect of the sensory brand experience within the brand environment, and also include physical attributes such as the texture, weight, and temperature felt by the consumer during brand interactions. For example, the texture of a sofa can elicit a tactile experience, which can convey the brand’s personality [104]. Unlike vision and hearing, haptic experience is more direct, and it can help consumers enhance their perception of the brand through actual physical contact, thus influencing their emotional response and purchase decision. The tactile sensation experienced when a consumer runs their hand over the grooves on a bottle, conveying a sensory meaning derived from touch, is increasingly recognized as a critical attribute of the brand experience [96]. Some of the sensations associated with touchpoints are enough to create a perception in the consumer’s mind, which builds a memorable brand experience and emotionally connects the consumer to the brand [105]. The link between touching a product and product evaluation was established in the study by Peck et al. [106]. Furthermore, Jha et al. [107] found that allowing a consumer to touch a product improves the consumer’s ability to interact with the product, thereby increasing the likelihood of purchase. However, tactile experience not only exists in direct contact with the product but can also indirectly affect consumers’ brand experience through product packaging, store design, etc. Krishna and Morrin [108] found that beautifully textured packaging designs can enhance consumers’ overall brand perception, which not only helps increase the brand’s attractiveness but also improves consumers’ purchase intention and brand loyalty. In luxury and high-end products, tactile perception often reinforces a brand’s high-end positioning and uniqueness through packaging and store displays [109]. In addition, Krishna and Morrin [108], in their study on the effect of the tactile sensation of product packaging on gustatory perception, pointed out that tactile imagery also affects the evaluation of products. On the other hand, Grohmann et al. [103] explored how tactile mechanisms can be used to enhance the quality of consumer evaluations of retail environments. In retail strategy, tactile is considered a vital sense that should be preserved because it provides consumers with a complete cognitive experience of the product, and even if the other senses are temporarily unavailable or in isolation, the tactile component can still play a vital role in the consumer’s perceptual process [102]. Tactility can influence consumers’ emotional responses, perceptions of authenticity, and purchase intentions through physical contact and sensory feedback in physical and digital environments. In summary, the following hypothesis can be made:

**Hypothesis 3 (H3)** Tactile mental imagery has a significant positive impact on brand experience.

### 2.5. Gustatory and brand experience

Gustation is a multisensory experience, and the taste on the tongue is only one aspect [110]. When considering brand experience, the gustatory component is often overlooked. However, in specific contexts, gustatory imagery not only influences our gustatory experience but also our portion size choices, enjoyment, and consumption [10]. Particularly in the restaurant and food industry, brands can stimulate consumers’ perceptions of taste and emotional experience through gustatory stimuli, thus enhancing their perception of the brand and its favorability [111]. Branded products with unique flavors can stand out more easily and create differentiation [112]. Thus, gustatory imagery can be elicited by emphasizing the multisensory experience in the consumption of food or beverages. Advertisements focusing on multisensory experiences can trigger thoughts and imagery about the taste of food, resulting in more positive and realistic taste evaluations than advertisements focusing on a single taste sensation alone [112]. A study by Elder et al. [87] found that taste requires more physical distance to be experienced, and consumers also feel closer psychologically. This psychological distance also affects the detail and color of imagery formed in the mind [98]. Studies have gradually begun to pay attention to the role of taste in brand experience, although they rarely mention it in isolation.

Khan and Fatma [113] found that gustation is crucial during brand experience. For example, tasting food or visiting a restaurant is closely related to the mutual perception process. Liu et al. [114] further pointed out that brands can create a unique and memorable gustatory experience by providing tasty food or beverages, which not only impress consumers but also trigger their emotional resonance, strengthen their emotional connection, and facilitate the establishment of brand loyalty. Similarly, Wiedmann et al. [115] pointed out that gustatory perception is closely related to consumers’ emotional responses, and a pleasant gustatory experience can trigger positive emotional resonance and enhance consumers’ favorability towards the brand. Gustation is unique in that its effect depends on parallel stimuli. When we feel that something tastes good, it is not only the taste bud receptors on the tongue that provide information, but receptors representing different sensory modalities are also involved [116]. This connection emphasizes that the pleasures of the table lie not only in the experience of oral taste but also in the resonance of the heart. Krishna [117] indicated that brands can not only provide essential sensory enjoyment through taste but can also be integrated with cultural storytelling through the design of the product’s flavor. This experience allows customers to feel the care and intention of the brand, which enhances their brand experience. Roggeveen et al. [118] indicate that gustatory cues are a critical factor in consumers’ perception of brand messages following a taste sampling experience. In recent years, an increasing number of brands have attracted consumers seeking personalized experiences by launching limited edition or customized flavors. This unique brand experience, shaped by the sense of taste, further strengthens the brand’s market competitiveness and consumer brand loyalty [119]. Moreover, combining taste with other sensory channels can give consumers a richer and more integrated brand experience [120]. For example, combining it with package design or brand ambiance can strengthen the brand image through consistent sensory signals, allowing consumers to form stronger brand memories during the experience. Although consumers cannot taste products directly in digital shopping environments, gustatory sensations still positively influence the brand experience through multisensory simulations and associations. With the development of e-commerce and digital technologies, brands are increasingly communicating the gustatory properties of their products virtually. Krishna and Schwarz [121] noted that visual and verbal cues could evoke consumers’ associations with taste in online shopping environments, indirectly enhancing their expectations of the product and brand experience.

Consumers’ exploration of multisensory cues in a brand environment affects their internal processing of the sensory brand experience. Taste plays a supporting role in this process, helping to realize synergies between the senses, thereby increasing the likelihood of customer acceptance of information and brand experience [92]. As the body interacts with touchpoints along the customer journey, it collects neurophysiological data on the gustatory cues provided by the brand. These data are then transmitted through neural circuits to the brain’s command center, where they are processed and evaluated, leading to a sensory brand experience [46]. This process not only assists consumers in building sensory experiences with the brand but also deepens their emotional connection to it, enhancing the depth and breadth of the brand experience. Based on the above theories, the following hypothesis is made:

**Hypothesis 4 (H4)** Gustatory mental imagery has a significant positive impact on brand experience.

### 2.6. Olfactory and brand experience

As a key sensory attribute, olfaction may be more effective in eliciting consumer responses than other modal (e.g., visual) stimuli [10]. Odors used in branded environments may induce pleasurable experiences and facilitate specific consumer responses [122]. Olfaction can quickly evoke emotions and memories and profoundly influence consumers’ subconscious experience of a brand [17]. Odors help to strengthen the persistence of a space in memory, thus enhancing the memorability and branding of that place [123]. In addition, odors enhance the visual recall of verbal information [124].

Roschk and Hosseinpour [125] found that the processing of olfactory signals occurs primarily in more primitive areas of the brain, which require fewer cognitive resources to automate behavior. Positive olfactory stimulation serves two essential purposes: eliciting pleasure and stimulating brand recall or information retrieval. Krishna and Elder [116] further noted a specific neural connection between the olfactory nervous system and the brain’s sensory cortex associated with emotional memory. This connection readily evokes emotional memories and responses to olfactory signals, enhancing the priming effect [126]. As the body interacts with touchpoints in the customer journey, it collects neurophysiological data on the olfactory aspects provided by the brand. These brand data are then transmitted through neural circuits to the command centers of the brain, where they are processed and evaluated, generating a sensory brand experience [46].

Olfaction plays a crucial role in facilitating sensory synergies that enhance the likelihood that the customer will be receptive to the message and the brand experience [92]. For example, when smelling a perfume, consumers not only perceive and remember the scent but may also develop internal imagery that includes visual, auditory, and tactile sensations that are also “experienced” [10]. Krishna et al. [127] found that incorporating visual images of food in advertisements effectively stimulated olfactory imagery. Due to the strong correlation between olfactory and gustatory senses, olfactory imagery directly influences people’s desire to consume food, and imagining the smell of food produces a physiological response similar to touching the food, thus enhancing the perception of authenticity. Particularly in the food industry, the aroma of food is essential in creating a positive experience [74]. When people imagine a particular odor, they construct this imagery through their olfactory sense. Interestingly, consumers have a weaker olfactory response when imagining harmful odors compared to positive odors [128]. Spence [129] found that the smell of food can deposit sensory markers in the brain, which can induce purchasing behaviors, prolong shopping time, and create a positive brand experience. Roy and Singh’s study [130] demonstrated that olfactory cues are an invaluable tool in retailing that can stimulate the assessment and prediction of consumer responses, influencing consumers’ olfactory perception and emotional experience and thereby enhancing their brand awareness and preferences.

In addition, olfaction can provide consumers with a richer and more integrated brand experience through integration with other sensory channels [114]. For example, in the restaurant industry, combining olfactory and gustatory senses can make the dining experience more profound and memorable for consumers [131]. Spangenberg et al. [88] emphasized the notion of ambiance. They tested a conditional multi-stimulus model in a Christmas environment to investigate the interactive effects of ambient Christmas scents and Christmas music on consumers’ evaluations of product and brand experiences. In the digital marketing of brands, although consumers cannot directly experience scents in virtual environments, some brands indirectly communicate the olfactory experience by combining visual and auditory cues. For example, Barbosa et al. [132] noted that brands could enhance the online shopping experience and brand appeal by using specific visual and verbal symbols to evoke olfactory associations in consumers. Errajaa et al.’s study [133] found that scent communication that is consistent with brand image helps to enhance brand experience by increasing customer satisfaction, repeat purchase intention, and perceptions of products and services. Based on the above theoretical basis, the following hypothesis can be made:

**Hypothesis 5 (H5)** Olfactory mental imagery has a significant positive impact on brand experience.

### 2.7. Autonomy and brand experience

Autonomy refers to an individual’s need for control and choice over their behavior [134] and is highly correlated with their brain power and ability to manipulate mental scenes [22]. Unlike intentional mental imagery, autonomous mental imagery is more spontaneous. For example, autonomy imagery may arise simply by viewing a picture, reading a specific word or description, or seeing an object or person [10]. Autonomy has been extensively studied in mental imagery as an essential theme in structural attributes. However, despite some existing research, more comprehensive studies on brand experience are needed [135]. Autonomy enhances consumers’ sense of control, thus increasing the positivity of the brand experience. Deci and Ryan’s Self-Determination Theory [136] states that individuals experience higher intrinsic motivation and pleasure when they feel the autonomy to make choices. This theory has been widely used in brand marketing research, suggesting that when consumers have more choices in brand interactions, they feel that personal values and brand fit are more vital, enhancing identification and emotional connection with the brand.

Satar et al. [137] found that the degree of consumer autonomy in brand experience is closely related to the perception of and engagement with the brand. When consumers feel empowered to make autonomous choices, they are more likely to interact with that brand on a deeper level, which enhances their perception and engagement. Schlosser [138] found that when participants could interact with the product autonomously online, they were more engaged in imagery and their real perceptions of the product were enhanced. This interaction enhanced not only participants’ attitudes toward the product but also their purchase intentions. Lunardo and Saintives’ study [139] suggested that brands can enhance consumers’ brand experience and emotional engagement by offering personalized products and services and brand activities that support autonomous choices, promoting emotional connections between brands and consumers. For example, some brands provide consumers with customization options that enable them to personalize their products according to their preferences and needs. This practice not only empowers consumers with more autonomy of choice but also enhances their brand experience. When consumers have autonomy, they may experience more happiness [139] because autonomy can positively contribute to enjoyment and well-being [140]. Kim and Lee [141] found that an essential psychological variable is the extent to which customers’ needs for autonomy are met. When brands encouraged customers to engage in activities that expressed their true identity, customers experienced an intrinsic desire for freedom of choice being satisfied, which increased their confidence and sense of autonomy and significantly increased their satisfaction with autonomy. Gilal et al.’s study [142] further showed that brands that satisfy consumers’ needs for autonomy enhance brand attachment, brand advocacy, and positive customer emotional responses. With the rise of e-commerce and online shopping platforms, an increasing number of brands have begun to empower consumers with more autonomy through personalized recommendations, customized services, etc. Platforms such as Etsy and Zazzle allow users to design and customize their goods freely, greatly enhancing their brand experience [143]. This trend is also reflected in digital products, such as personalized settings and controls in industries such as software and gaming, which significantly enhances user satisfaction and perceptions of brand authenticity.

In the context of brand journeys, consumers whose need for autonomy is satisfied will show more loyalty [134]. Customers can independently select products and services of interest and enhance their sense of control through personalized settings and interactive engagement, thus enhancing their overall brand experience [134]. In summary, the following hypothesis can be made:

**Hypothesis 6 (H6)** Autonomy mental imagery has a significant positive impact on brand experience.

### 2.8. Spatial and brand experience

Spatial plays a crucial role in mental imagery, connecting all sensory attributes and bridging structural and intellectual attributes. Distance, direction, and location are the three main elements of the spatial component. Analysis shows that the distance between entities and observers is closely related to the distance between entities. The sense of orientation is easily disturbed when losing focus or trying to recall an image. However, with varying levels of attentional focus, individuals may be able to resist distortion or loss of sense of direction [22]. Spatial is crucial in brand experience, as it influences consumer perception, emotion, and behavior [144].

Kosslyn et al. [145] revealed important spatial properties of mental imagery, leading to the quasi-drawing theory (also known as description theory) of mental imagery. Spatial not only refers to the physical environment but also to a platform for brands to establish meaningful interactions with consumers and a medium to enhance brand awareness and foster emotional connections. The environment or design elements as a behavioral subject can influence the sensory brand experience in the physical environment [118]. Using fonts, colors, or images as actors affects the brand experience in virtual environments [146]. Donovan et al. [147] describe retail environments as emotional-psychological spaces, directing attention to the impact of the environment on consumer intentions and consumption behaviors. By integrating interior design elements, sensory stimuli, and experiential components, brands can effectively communicate their identities, values, and messages to create a unique and engaging brand experience. Kalantari et al. [148] found that attractive store layouts and environmental designs can enhance consumers’ emotional experience, purchase intentions, and brand perceptions and trust. By creating a comfortable and pleasant atmosphere, brands can shape consumers’ experience and enhance their image and competitive advantage. Since a brand’s spatial environment is co-created by the meaning creator and the consumer’s subjective consciousness, what manifests itself as the brand spatial environment in the consumer’s mind is usually more than what is expected. Brand spatial environments are dynamic, ever-changing, and constantly being reconfigured and redescribed through the (both intentional and unintentional) interventions of different types of actors to enable and constrain the outcome of the brand experience [46].

During the brand experience, the spatial design, arrangement, color, and overall context that customers are exposed to can impact their emotions and behaviors. Zha et al. [46] found that the spatial design and interiors of shopping malls are carefully planned to evoke spatial associations with a specific place in the memory of an individual or group. This spatial is not just a physical environment but is also a carefully curated historical and cultural narrative whose cultural power moves consumers in desired ways, triggering an emotional response to the retail space and influencing the emotional response evoked by a brand [149]. Venkatraman and Nelson [150] showed that when consumers engage with the spatial layout, colors, décor, and overall scenario, they reinterpret and create their own unique and meaningful esthetic experience. Smink et al. [151] found that spatial presence in virtual brand experience scenarios is a subjectively perceived construct that explains how virtual objects or environments are experienced as actual physical objects or environments, and perceived spatial presence increases purchase intentions. Although consumers cannot physically experience the physical environment, brands can create a simulated experience through the design and arrangement of virtual spaces that allows consumers to feel the presence and value of the brand, and can enhance the perceived authenticity of the brand through a personalized, immersive experience [152]. Park and Lee [153] found that applying augmented reality technology to online stores can enhance customers’ sense of immersion and presence, enhancing their brand experience. Whether shopping online or offline, customers reconstruct the spatial sensations they once experienced in the brand experience [22]. In summary, the following hypothesis can be made:

**Hypothesis 7 (H7)** Spatial mental imagery has a significant positive impact on brand experience.

### 2.9. Kinesthetic and brand experience

Kinesthetic is a sensory system used to determine the properties of objects interacting with the body, such as weight or stiffness. It needs to be combined with inputs from visual, tactile, and vestibular organs to help us interpret our environment [154]. Indeed, the kinesthetic component shows high correlations with the auditory, visual, and tactile components, suggesting that the motor–sensory cortex utilizes all three sensory properties to create mental imagery of the “kinesthetic” property [22]. In the mental imagery literature, motion is a more general term that can be translated as a kinesthetic property [86], referring to how an individual imagines interacting with an object [62]. Kinesthetic, as a sensory channel related to action and movement, can stimulate consumers’ bodily perception and enhance their emotional connection with and cognitive experience of the brand. The above three structures (i.e., spatial, autonomy, and kinesthetic) represent the structural components in the OMI model [22].

Zeng et al. [155] found that dynamic brand activities and physically interactive experiences can enhance consumers’ engagement and emotional experience, thus deepening their perception and favorability of the brand. For example, during the shopping process, consumers’ kinesthetic experiences, such as trying on, trying out, and test driving, will directly affect their evaluation of the product and their perception of the brand image. One study [62] found that pointing an object toward one’s dominant hand rather than away from it reinforces the mental imagery of interacting with that object, enhancing purchase intentions. Through actual physical actions (e.g., touching, trying on, or handling), consumers can better understand the product’s features and perceive its authenticity, leading to a more positive emotional response and brand experience [156]. For example, consumers’ evaluation of brand quality and performance can be enhanced through kinesthetic perception when operating equipment in an electronics showroom or trying sports equipment in a sports brand store. In addition, by designing innovative and fun interactive experiences, brands can effectively capture consumers’ attention and build a positive brand image and emotional connection.

Karampournioti and Wiedmann [157] found that storytelling using parallax motion effectively conveyed brand-related associations in consumers’ minds, enhanced their attitudes toward the brand, and increased their willingness to pay a higher price. Salazar et al. [158] demonstrated that consumers often request samples before making a purchase, highlighting the importance of kinesthetic stimuli and illustrating buyers’ sensitivity to associating products with emotional stimuli. The kinesthetic experience is a critical factor driving the pleasure experienced in virtual shopping. It can stimulate the three mental states of the body schema of online consumers, thus enhancing the pleasure of the brand experience [159]. Kinesthetic interactions in virtual reality simulate real tactile and motor experiences and enhance consumers’ sense of immersion and engagement, allowing their brand experience and perception of authenticity in a digital environment to be similar to those in a physical environment [160]. Such interactions, enhanced by virtual kinesthetic sensations, not only improve the technological and innovative aspects of the brand but also stimulate consumer interest and loyalty.

Therefore, brands can create more meaningful and memorable interactions with consumers, deepen the impression of brand personality, improve brand image recognition, and help create a more immersive and authentic brand experiences through kinesthetic in physical and digital environments,. Based on the above theoretical basis, the following hypothesis can be made:

**Hypothesis 8 (H8)** Kinesthetic mental imagery has a significant positive impact on brand experience.

### 2.10. Brand experience and brand authenticity

Holbrook and Hirschman [16] first introduced the concept of experience in consumer perception, initiating the search for related research. Andreini et al. [45] conducted a comprehensive review of the literature related to “brand experience.” They found that almost all of the research relied on the perspectives of brand experience theory scholars such as Schmitt and Brakus. Schmitt [17] conceptualized brand experience for the first time. Brakus et al. [18] further defined brand experience as “subjective, intrinsic consumer responses (including sensory, emotional, and cognitive) and behavioral responses evoked by experiential attributes associated with a brand when interacting with, shopping for, and consuming the brand”. Customers can experience a brand in various contexts, including direct and indirect interactions [161]. According to Iglesias et al. [32], direct interactions refer to customers purchasing, consuming, or using a brand’s products or services, while indirect interactions refer to customers perceiving a brand’s advertisements, marketing communications, word-of-mouth testimonials, news reports, and reviews. Any stimuli associated with a brand, such as brand image, environment, and communication methods, can trigger subjective responses from customers, thus affecting the overall brand experience [30]. More specifically, when customers feel pleasure and satisfaction when exposed to a brand, they internally develop positive feelings, attitudes, and perceptions about the brand; these result from an internal experience triggered by external exposure [162]. According to Shamim and Mohsin Butt [163], customers’ encounters with a brand’s marketing mix play a key role in brand experience, and positive or negative evaluations of these interactions directly affect their perceptions of brand credibility. Meanwhile, brand experience is a central factor in enhancing brand authenticity, effectively prompting customers to form a more favorable impression of the brand [164]. Although many scholars have explored brand experience from different perspectives and deepened their understanding of the phenomenon [15], the study of brand experience remains relatively new [46].

In addition, many scholars have worked on studying consumers’ preferences for authentically branded products to enhance their brand experience [23]. Modern consumers crave authenticity in all domains, and this quest for authentic brand experiences is further evidence of the critical role of brand authenticity in developing loyal consumers [165]. According to Campagna et al. [1], brand authenticity can be defined as natural brands with a distinctive style and an openness to honest consumer care that can withstand the test of time and trends. One study by Fritz et al. [26] suggests that brand authenticity involves perceived consistency in brand behavior that reflects its core values and norms, as a result of which the brand is perceived as being true to itself and not compromising its essence. Another study by Dwivedi and McDonald [28] found that authenticity is the trait and ability of brands to be consistent in what they say and do, reflecting the belief that brands possess the character trait of delivering on their promises [14]. When a brand’s attributes are reflected in its quality promise, heritage, and genuine behavior, consumers perceive the brand as delivering on its promise, i.e., embodying brand authenticity [27,28], thus enjoying a positive brand experience [166]. Given the heightened customer demand for brand authenticity, understanding it is critical to strengthening brand–customer engagement [167]. Fritz et al. [26] found that brand authenticity helps foster stronger emotional relationships between brands and customers. It can also fulfill consumer perception needs [27]. Another study by Gilmore and Pine [24] found that brand authenticity is emerging as an important purchasing criterion for consumers over product price and quality. It is becoming increasingly important to consumers’ purchasing decisions and brand interaction behaviors, and interest in this vital attribute is also growing [1]. Establishing brand authenticity has become an essential task for marketers, and brand authenticity as a differentiator will likely shape the marketing landscape for years to come [28]. Furthermore, some scholars have noted that future research should explore the role and path of brand experience in brand authenticity [168].

Lemon and Verhoef [13] emphasize creating sensory and emotional connections with consumers rather than just focusing on the physical characteristics of products and services. Recently, brand authenticity has overtaken quality as the primary purchasing criterion of consumer concern [24]. Consumers continue to seek unique and pleasurable experiences, which they want to enjoy during and after consumption through profound and positive memories [18,169]. These experiential elements are deliberately implanted in the customer’s memory to create positive emotions for the brand, such as love and passion [30]. A study by Bolton et al. [170] demonstrated how small, unobtrusive behaviors in a service environment can accumulate in the customer’s memory as unforgettable sensory states, thereby distinguishing one service from another. Furthermore, Brakus et al. [18] also noted that the intensity of a brand experience affects consumers’ ability to surmise the brand’s personality, affecting brand satisfaction and loyalty. Better brand performance will result in a positive brand experience for consumers, enhancing brand authenticity [24] and inspiring love for the brand [27]. Jian et al. [171] stated that brand authenticity is directly dependent on the consumer’s actual experience. According to Rodrigues et al. [9], customers’ growing desire for brand experience emphasizes the importance of brand authenticity in fostering positive customer interactions. Customers’ brand experience is seen as a driver of enhanced brand authenticity, directly leading to a more positive perception of the brand [164]. Safeer et al. [27] also examined this relationship, finding that brand experience has a positive role in enhancing brand authenticity. On the other hand, Robbins et al. [172] stated that consistency across the dimensions of brand experience helps establish brand authenticity, as it is a crucial outcome of brand experience that can drive brand prosperity and sustainability. In addition, Park et al. [14] conducted a study in an e-commerce environment during the COVID-19 pandemic and found that a positive brand experience significantly affects brand authenticity. Building on this foundation, our study expands and deepens the research in several ways. First, our research context is not limited to the pandemic period, nor does it only focus on online shopping. It covers various shopping scenarios, including offline physical and multichannel shopping experiences. Through this expansion, we can explore the impact of brand experience on brand authenticity in different shopping contexts more comprehensively, thus enhancing the applicability and generalizability of this study. Second, regarding the research variables, we introduced the OMI as a dependent variable to further refine the analysis of brand experience. We explored how different sensory and structural attributes affect brand authenticity through brand experience. This refinement not only deepens the understanding of the relationship between brand experience and brand authenticity but also reveals the critical role of the OMI in this process, providing a new perspective for academic research in related fields. Therefore, the following hypothesis can be made:

**Hypothesis 9 (H9)** Brand experience has a significant positive impact on brand authenticity.

### 2.11. Moderating role of social presence

The concept of social presence is based on social presence theory, which details the ability of communication media to convey social cues [173]. Short et al. [39] define social presence as “the salience of others in mediated communication and the consequent salience of interpersonal interactions”. Social presence describes the visibility of social cues in mediated communication and whether or not these cues are effectively conveyed in the medium compared to direct face-to-face interactions. This is further emphasized by Fulk et al.‘s [174] definition of the extent to which the medium makes the presence of others felt by the user. Social presence theory also states that the effectiveness of social presence is the basis for successful interaction and communication between parties in a technological medium. Research has shown that social presence facilitates online interactions and promotes social commerce [175]. Akram et al. [176] found that social presence has recently been widely used in e-commerce. For example, Shi et al. [40] found that social presence affects consumers’ impulse buying behavior, in an e-commerce environment. Chen et al. [41] also found that social presence affects consumers’ purchase decisions and purchase intentions. In addition, social presence has been used to compensate for the lack of human assistance and physical distance in online website systems [177], helping individuals shorten the social time required to bring the communicating parties closer together and enhancing users’ perceptions of others during virtual activities [178]. Therefore, in social commerce, social presence is defined as “the degree to which customers establish personalized, warm, intimate, and social interactions with others in a social commerce environment” [179]. In other scenarios, the experience of such interactions has been described as a sense of psychological closeness, comfort, and togetherness when interacting with others in a mediated environment [180]. One study by Tsai et al. [43] found that interacting and communicating with a customer service bot with social presence increased customer engagement with the brand experience. Social presence as a technology-mediated artifact can convey a sense of human warmth and socialization [181].

In online environments, social presence is perceived in terms of social elements [44]. It can be perceived in various ways, such as through texts rich in social elements [182] and by describing real-time online chat interactions [44]. Short clips, emoticons, and animations can also be included in addition to text, allowing users to manipulate how others perceive their social presence [183]. Social presence has been recognized as an essential concept in interface design for studying online web systems to overcome the barriers created by a lack of human assistance and physical distance [177]. Daliri et al. [184] found that customers in online shopping situations are more likely to perceive social presence. Lu et al. [185] also found that when individuals make purchase decisions, they are directly influenced by social interaction. However, people can perceive social presence without direct interaction or interpersonal relationships [186].

Another study by Quester et al. [187] found that consumers express their sense of belonging in society by purchasing brands, fulfilling their need for self-identification [188]. Consumers tend to judge the authenticity of brands through the lens of social presence. Earlier studies have found that social presence significantly impacts customers’ trust in brands because they view others as possible purchasers [189,190]. Hassanein and Head [191] state that users are more likely to have interpersonal interactions, perceived support, and trust when they perceive the presence of others in a virtual environment. Customers usually learn more about a product or service through customer feedback in online shopping environments with a social presence. When feedback from others is positive, customers tend to be more willing to trust the service or product because influence from people like them proves more effective [189]. This increases consumer trust, reduces purchase uncertainty, and helps customers reduce the risks associated with online shopping [192], with feedback and reviews resulting from a brand’s social presence verifying that the brand is authentic and trustworthy, thus enhancing perceived brand authenticity due to trust [193]. Social presence conveys a sense of warmth and trustworthiness between people [181], and brands can further solidify this trust through authentic social interactions. As a result, customers are not only willing to make repeat purchases, but are also more likely to actively participate on social commerce platforms, join online brand communities, and deepen their interactions and connections with brands.

In addition to its impact on brand authenticity, social presence positively affects brand experience. Osei-Frimpong and McLean [194] found that social presence can significantly influence consumers’ perceptions and behaviors during their experience with a brand through mechanisms such as social influence and social comparison. Furthermore, a study by Cyr et al. [195] showed that introducing social presence in e-shopping scenarios improved customers’ attitudes and behavioral intentions. In addition to preventing disengagement from a brand’s online presence resulting from positive offline behavior, social presence also has a motivational effect [196]; it can enhance customers’ positive attitudes and purchase intentions toward products and brands [14]. It can also generate a stronger sense of presence, which translates into an enhanced brand experience [57]. This suggests that social presence narrows the perceived gap between participants and brings about a degree of closeness, which also depends on the richness of media messages [197]. Social presence enables people to feel in touch with or understand another person’s intentions, perceptions, or emotional states [198]. The relative proximity and number of other customers can influence a customer’s shopping experience, even in non-interactive social situations [199]. In addition, De Regt et al. [200] found that emotional involvement in the brand experience is higher when customers perceive social presence. Consumers establish emotional connections through social interactions, enhancing social presence and increasing intimacy. Enhanced social presence enables consumers to effectively communicate immediacy or non-immediacy through non-verbal (e.g., physical proximity, images, and facial expressions) and verbal means [201]. In addition, it provides a social context and support for such emotional connections, giving more depth and meaning to the brand experience [183]. According to Park et al. [14], enhancing social presence strengthens the link between brand experience and authenticity. Therefore, given these ample findings, the following hypothesis can be made:

**Hypothesis 10 (H10)** Social presence plays a moderating role between brand experience and brand authenticity.

### 2.12. Theoretical model

**Fig 1:**
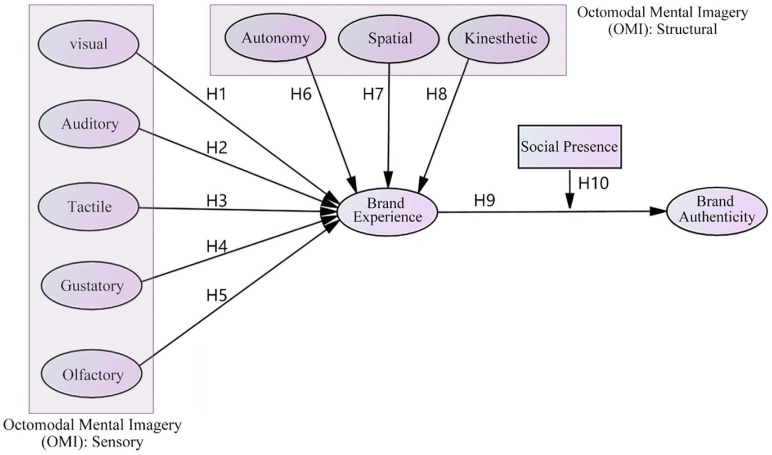
Research model.

The theoretical framework of this study is shown below (Fig 1). The model is built around brand experience and brand authenticity. It explores the impact of OMI on brand experience (H1−H8), the impact of brand experience on brand authenticity (H9), and the moderating role of social presence in the relationship between brand experience and brand authenticity (H10).

## 3. Materials and methods

### 3.1. Development of research instruments

In this study, a closed-ended questionnaire was used to collect data, in which respondents were asked to rate each statement according to the format of a seven-point Likert scale (in which seven points indicate strong agreement and one point indicates strong disagreement) [202]. In addition to measuring various variables, the questionnaire also included information on demographic characteristics.

The present study identified 47 items to be measured in the questionnaire, and the scales used were selected and adapted from existing well-established scales, as shown in S1 Table. Among these, the scales used to measure the sensory (visual, auditory, tactile, gustatory, and olfactory) and structural properties (autonomy, spatial, and kinesthetic) of OMI were adopted from Khalilzadeh et al. [22]. The scale measuring brand experience was adapted from Brakus et al. [18]. The scale for the dependent variable of brand authenticity was adapted from Schallehn et al. [166]. The scale for the moderator variable of social presence was adapted from Lu et al. [185].

### 3.2. Sampling and data collection

In our study, sample selection was performed using a blend of simple random sampling and purposive sampling. Our goal was to ensure that diverse demographic characteristics were represented in the population under investigation to guarantee an adequate sample size capable of detecting substantial effects and attaining statistical significance. The aim was to mitigate potential bias and enhance the generalizability of our findings. Sample size determination was guided by statistical considerations and the nature of the study objectives.

The questionnaire for this study was designed, created, and collected using Sojump’s online survey platform. The link/QR code for the electronic questionnaire was distributed via email, WeChat, and SMS. In order to motivate the respondents and stimulate authentic responses, this questionnaire offered bonus reward of a random amount (distributed after review). In addition, Sojump is equipped with various verification mechanisms, such as IP address, time of completion, and prevention of duplicate submissions, which allowed us to ensure that the results were not interfered with by duplicate data and malicious fillings. Therefore, Sojump is a trustworthy tool for conducting data research and evaluation and is widely used in various research fields [203].

This study selected a new-style tea beverage brand as the object of analysis based on its extensive market coverage and high brand recognition in China, which provides strong background support for studying consumers’ brand experience. The research context covers online and offline integrated environments to reflect the current diversified shopping styles and meet consumers’ brand experience needs in different channels, aiming to analyze in depth consumers’ perceptions of brand experience. The survey includes several indicators, such as OMI, brand experience, and its impact on brand authenticity, as shown in S1 Table. The survey process was conducted in strict accordance with the research design to ensure the reliability and validity of the data. In order to improve the accuracy of this study, minimize brand awareness bias, and ensure the representativeness of the data, we chose respondents who had a certain degree of familiarity with the new-style tea drink brand and set up a brand awareness screening in the initial part of the questionnaire to exclude respondents who were unfamiliar with it.

As the scales used in this study involve marketing and psychology terminology; the original versions are all in English. Hence, they needed to be translated into Chinese for the respondents. In order to ensure the reliability and comprehensibility of the questionnaire, a small and generally representative pre-survey first invited 148 people to this study [204]. After the survey, the researcher followed up with the subjects via the contact information they had provided, checked the linguistic structure and readability of the questionnaire content [205], and made adjustments to improve and embellish the questionnaire based on the feedback. Finally, multilingual experts compared the questionnaires before and after translation, and the results showed that, except for minor adjustments to the order and use of specialized terminology in different contexts, the translated Chinese version remained consistent with the measurements of the original English version.

As this study involved human participants, a written informed consent form was provided on the first page of the questionnaire. Anonymity and confidentiality were ensured by the fact that no personal names were asked for when completing the questionnaire, with identification numbers being used instead. Moreover, the questionnaire was only administered after clicking the “Agree” button, and participants could withdraw at any time during their completion of the questionnaire. Clicking the “Disagree” button resulted in an immediate exit from the questionnaire. According to the regulatory policy for the location where the data were collected, no ethical review was required for this study. However, we provided relevant ethical review materials. Additionally, it should be noted that minors were not included in this study. The researchers officially distributed the questionnaires on 7 August 2023, and 578 questionnaires were collected by 12 September 2023. In order to improve the quality of data and to ensure the authenticity of the subjects, the questionnaire also contained fixed-option distractor items: “Please select the last option in this question (Strongly Agree).” Based on previous studies by Oppenheimer and Tourangeau et al. [206,207], it can be assumed that those who did not select the item did not double-check what the question was asking. Following this, and combining the multiple validation mechanisms of the Sojump system, a total of 150 invalid questionnaires were deleted and 428 valid questionnaires were obtained, with a validity rate of 74%. Nunnally and Bernstein [208] recommended a sample size of at least 300. At the same time, Charter and Polonsky [209] concluded that a sample size of at least 400 was required to estimate Cronbach’s Alpha coefficient with sufficient precision. The sample size was established considering a 10% confidence level and a 15% margin of error. This project’s sample size is therefore sufficient and appropriate for meeting this study’s requirements [210,211].

### 3.3. Research application used

According to Hair et al. [212], the internal consistency and validity of measurement data can be analyzed and verified through reliability, exploratory factor analysis (EFA), correlation, average variance extraction (AVE), and confirmatory factor analysis (CFA). We employed the two-step method measurement approach introduced by Anderson and Gerbing [213].

In this study, statistical data analysis was conducted using SPSS 25.0. Specifically, descriptive statistics were analyzed for demographic variables, and normality tests, reliability analyses, and validation factor analyses were performed on the collected data. In addition, stratified regression analyses were conducted to test the moderating effect of social presence on the relationship between brand experience and brand authenticity. We employed structural equation modeling (SEM) with AMOS 23.0 to investigate the relationship between the latent variables, and the model was modified by adding a two-by-two correlation between the independent variables and testing the research hypotheses in conjunction with path analysis.

## 4. Results

### 4.1. Descriptive statistics

The primary demographic information of the survey sample in this study is described in Table 1.

**Table 1:** Basic demographic information.

Items	Categories	Sample Size	Percentage (%)	Cumulative Percentage (%)
Gender	Male	203	47.43	47.43
	Female	225	52.57	100.00
Age	18–25 years old	73	17.06	17.06
	26–35 years old	136	31.78	48.83
	36–45 years old	133	31.07	79.91
	Over 45 years old	86	20.09	100.00
Marital status	Single	162	37.85	37.85
	Married	266	62.15	100.00
Education level	Junior high school or below	5	1.17	1.17
	High school or technical secondary school	22	5.14	6.31
	Junior college	143	33.41	39.72
	Undergraduate	161	37.62	77.34
	Master’s degree or above	97	22.66	100.00
Average monthly income (including allowances, dividends, and other forms of income)	CNY 5000 and below	42	9.81	9.81
	CNY 5001–10,000	181	42.29	52.10
	CNY 10,001–20,000	176	41.12	93.22
	CNY 20,001–30,000	18	4.21	97.43
	CNY 30,001–50,000	7	1.64	99.07
	CNY 50,001 and above	4	0.93	100.00
Total		428	100.0	100.0

It was found that 47.43% of the respondents identified as men, and 52.57% identified as women. Regarding age, 31.78% of the respondents were between 26 and 35 years old. In terms of marital status, 37.85% of respondents identified as single, and 62.15% identified as married. Regarding education level, 37.62% of respondents had a bachelor’s degree and 22.66% of respondents had a master’s degree or above. In terms of average monthly income, 42.29% of respondents earned between CNY 5001 and 10,000.

By analyzing the above data, some preliminary conclusions were drawn: the proportion of women was slightly higher than that of men, the age was mainly concentrated in the range of 26–45 years old, the proportion of married people was higher, the education level of most of the people was bachelor’s degree and above, and the income level was mainly concentrated in the range of CNY 5001–20,000. Participants of all ages, genders, marital statuses, education levels, and average monthly incomes in the questionnaire were involved in the survey, indicating that the survey sample was relatively comprehensive and met the needs of the study.

### 4.2. Reliability analysis

According to the study of Hair, Cronbach’s Alpha was used to measure the scale’s reliability [214,215]. As can be seen from the statistical results (Table 2), the Cronbach’s Alpha values for the 11 dimensions designed for this study ranged between 0.832 and 0.877, which all exceeded the threshold of 0.7; this indicates that the internal consistency of the questionnaire dimensions was high [216]. Therefore, the data collected in this survey passed the test in terms of reliability.

**Table 2:** Reliability statistics.

Themes	Constructs	No. of Items	Sample Size	Cronbach’s α
OMI (sensory property)	Visual	4	428	0.847
	Auditory	4	428	0.848
	Tactile	4	428	0.849
	Gustatory	4	428	0.855
	Olfactory	4	428	0.832
OMI (structural property)	Autonomy	4	428	0.854
	Spatial	4	428	0.837
	Kinesthetic	4	428	0.835
Brand experience	Brand experience	6	428	0.873
Brand authenticity	Brand authenticity	5	428	0.877
Social presence	Social presence	4	428	0.866

### 4.3. Normality testing

According to Hair et al. [215], data normality is a crucial assumption when using SEM models (especially those on AMOS). It is necessary to performing normality tests on the data and decide whether the data needs to be transformed based on the results to ensure that the model estimates are reliable. Considering the results of the normality testing (Table 3), the means of the measurement items ranged from 4.322 to 4.82, which could be considered a medium level overall. The standard deviation was relatively low, meaning the data for each measurement item were relatively concentrated and less discrete. The absolute skewness value was below 3 for most variables, and the absolute kurtosis value was less than 10. According to McDonald and Ho [217], this indicates that the measured data meet the requirements of normal distribution.

**Table 3:** Normality testing.

Themes	Constructs	Items	Sample Size	Mean	Std.	Skewness	Kurtosis
OMI (sensory property)	Visual	VIS1	428	4.533	1.586	−0.205	−0.676
		VIS2	428	4.451	1.535	−0.059	−0.695
		VIS3	428	4.685	1.898	−0.478	−0.942
		VIS4	428	4.493	1.555	−0.195	−0.633
	Auditory	AUD1	428	4.355	1.527	−0.185	−0.448
		AUD2	428	4.643	1.851	−0.468	−0.879
		AUD3	428	4.472	1.538	−0.195	−0.471
		AUD4	428	4.488	1.531	−0.149	−0.503
	Tactile	TAC1	428	4.743	1.801	−0.539	−0.647
		TAC2	428	4.444	1.553	−0.053	−0.73
		TAC3	428	4.449	1.598	−0.273	−0.546
		TAC4	428	4.481	1.54	−0.214	−0.424
	Gustatory	GUS1	428	4.449	1.601	−0.216	−0.615
		GUS2	428	4.509	1.536	−0.248	−0.465
		GUS3	428	4.456	1.589	−0.239	−0.6
		GUS4	428	4.759	1.884	−0.546	−0.795
	Olfactory	OLF1	428	4.82	1.787	−0.631	−0.51
		OLF2	428	4.512	1.542	−0.284	−0.463
		OLF3	428	4.561	1.49	−0.12	−0.628
		OLF4	428	4.481	1.492	−0.197	−0.422
OMI (structural property)	Autonomy	AUT1	428	4.53	1.54	−0.152	−0.625
		AUT2	428	4.465	1.552	−0.174	−0.612
		AUT3	428	4.673	1.795	−0.556	−0.663
		AUT4	428	4.495	1.598	−0.24	−0.731
	Spatial	SPA1	428	4.815	1.799	−0.623	−0.569
		SPA2	428	4.551	1.55	−0.18	−0.634
		SPA3	428	4.621	1.542	−0.313	−0.524
		SPA4	428	4.435	1.45	−0.212	−0.314
	Kinesthetic	KIN1	428	4.706	1.822	−0.422	−0.844
		KIN2	428	4.528	1.438	−0.123	−0.453
		KIN3	428	4.437	1.504	−0.053	−0.579
		KIN4	428	4.397	1.518	−0.095	−0.673
Brand experience	Brand experience	BE1	428	4.404	1.388	0.052	−0.503
		BE2	428	4.402	1.443	−0.125	−0.624
		BE3	428	4.332	1.484	−0.052	−0.702
		BE4	428	4.423	1.395	−0.041	−0.461
		BE5	428	4.432	1.402	−0.032	−0.597
		BE6	428	4.633	1.632	−0.39	−0.73
Brand authenticity	Brand authenticity	BA1	428	4.418	1.573	−0.139	−0.632
		BA2	428	4.409	1.596	−0.153	−0.601
		BA3	428	4.386	1.565	−0.166	−0.607
		BA4	428	4.36	1.546	−0.246	−0.356
		BA5	428	4.638	1.952	−0.436	−0.959
Social presence	Social presence	SP1	428	4.757	1.798	−0.591	−0.675
		SP2	428	4.582	1.496	−0.129	−0.705
		SP3	428	4.505	1.537	−0.329	−0.464
		SP4	428	4.491	1.56	−0.206	−0.637

### 4.4. Common method bias test

According to the results of the Harman one-way test and exploratory factor analysis used in this paper, the data analysis of the variance explained rate (Table 4) revealed that the variance explained rate of the first common factor was 31.277%, which was lower than the threshold of 40%. According to the criteria of Harman’s one-way test, when the variance explained rate of the first common factor is lower than 40%, it can be concluded that there is no substantial common method bias. Therefore, there was no serious common method bias in this study.

**Table 4:** Variance explained rate.

Total Variance Explained									
Component	Initial Eigenvalues			Extraction Sums of Squared Loadings			Rotation Sums of Squared Loadings		
	**Total**	**% of Variance**	**Cum.%**	**Total**	**% of Variance**	**Cum.%**	**Total**	**% of Variance**	**Cum. %**
1	14.700	31.277	31.277	14.700	31.277	31.277	3.527	7.504	7.504
2	2.405	5.118	36.395	2.405	5.118	36.395	3.245	6.904	14.408
3	2.195	4.670	41.065	2.195	4.670	41.065	2.953	6.284	20.692
4	2.025	4.308	45.373	2.025	4.308	45.373	2.948	6.273	26.965
5	1.897	4.036	49.408	1.897	4.036	49.408	2.905	6.181	33.146
6	1.801	3.831	53.240	1.801	3.831	53.240	2.883	6.134	39.280
7	1.785	3.799	57.038	1.785	3.799	57.038	2.880	6.129	45.409
8	1.688	3.592	60.630	1.688	3.592	60.630	2.844	6.052	51.461
9	1.477	3.143	63.774	1.477	3.143	63.774	2.786	5.928	57.388
10	1.310	2.787	66.561	1.310	2.787	66.561	2.778	5.910	63.298
11	1.173	2.495	69.056	1.173	2.495	69.056	2.706	5.757	69.055

### 4.5. Confirmatory factor analysis

#### 4.5.1. Convergent validity.

According to the results (Table 5), the factor loading coefficients of the 11 factors analyzed in this study were greater than 0.6, and the corresponding AVE values were greater than the recommended level of 0.5 [214]. Furthermore, the analysis results indicated that all factors had CR values exceeding 0.7; this means that the data analyzed in this study performed well regarding convergent validity [218].

**Table 5:** Convergent validity statistics.

Themes	Constructs	Items	B	S.E.	C.R.	*p*	β	AVE	CR
OMI (sensory property)	Visual	VIS1	1	–	–	–	0.766	0.590	0.852
		VIS2	0.893	0.062	14.31	***	0.707		
		VIS3	1.308	0.077	16.902	***	0.837		
		VIS4	0.97	0.063	15.392	***	0.758		
	Auditory	AUD1	1	–	–	–	0.74	0.591	0.852
			AUD2	1.378	0.085	16.278	***	0.842	
			AUD3	1.024	0.069	14.761	***	0.753	
			AUD4	0.995	0.069	14.416	***	0.735	
	Tactile	TAC1	1	–	–	–	0.842	0.592	0.853
			TAC2	0.725	0.047	15.415	***	0.709	
			TAC3	0.808	0.048	16.971	***	0.767	
			TAC4	0.765	0.046	16.614	***	0.754	
	Gustatory	GUS1	1	–	–	–	0.767	0.603	0.858
			GUS2	0.931	0.061	15.188	***	0.744	
			GUS3	0.977	0.063	15.418	***	0.755	
			GUS4	1.282	0.075	17.023	***	0.836	
	Olfactory	OLF1	1	–	–	–	0.815	0.561	0.836
			OLF2	0.781	0.051	15.376	***	0.738	
			OLF3	0.722	0.049	14.646	***	0.706	
			OLF4	0.753	0.049	15.302	***	0.734	
OMI (structural property)	Autonomy	AUT1	1	–	–	–	0.731	0.598	0.856
		AUT2	1.039	0.071	14.579	***	0.753		
		AUT3	1.31	0.083	15.756	***	0.822		
		AUT4	1.115	0.074	15.16	***	0.785		
	Spatial	SPA1	1	–	–	–	0.804	0.568	0.840
			SPA2	0.813	0.052	15.729	***	0.759	
			SPA3	0.805	0.051	15.656	***	0.756	
			SPA4	0.691	0.049	14.182	***	0.69	
	Kinesthetic	KIN1	1	–	–	–	0.783	0.564	0.838
			KIN2	0.723	0.05	14.568	***	0.718	
			KIN3	0.781	0.052	15.065	***	0.741	
			KIN4	0.809	0.052	15.481	***	0.761	
Brand experience	Brand experience	BE1	1	–	–	–	0.707	0.538	0.875
		BE2	1.01	0.076	13.355	***	0.687		
		BE3	1.13	0.078	14.491	***	0.748		
		BE4	1.034	0.073	14.125	***	0.728		
		BE5	1.045	0.074	14.198	***	0.732		
		BE6	1.323	0.086	15.379	***	0.796		
Brand authenticity	Brand authenticity	BA1	1	–	–	–	0.745	0.597	0.881
		BA2	1.001	0.067	14.956	***	0.735		
		BA3	1.008	0.066	15.385	***	0.754		
		BA4	0.991	0.065	15.304	***	0.751		
		BA5	1.454	0.082	17.801	***	0.872		
Social presence	Social presence	SP1	1	–	–	–	0.815	0.621	0.868
		SP2	0.765	0.047	16.29	***	0.749		
		SP3	0.854	0.048	17.938	***	0.814		
		SP4	0.824	0.049	16.931	***	0.774		

***p < 0.001.

#### 4.5.2. Discriminant validity.

The bold numbers on the diagonal (Table 6) are the AVE square root values; the remaining numbers are the correlation coefficients. For the visual construct, the AVE square root value of 0.768 was greater than the maximum value of 0.505 for the absolute value of the correlation coefficients between the factors, which means that it had good discriminant validity [219]. Similarly, the discriminant validity of the other constructs was better. Therefore, the scales in this study had good discriminant validity.

**Table 6:** Discriminant validity results.

		Vis-	Aud-	Tac-	Gus-	Olf-	Aut-	Spa-	Kin-	BE	BA	SP
OMI (sensory property)	Visual	**0.768**										
	Auditory	0.387	**0.769**									
	Tactile	0.316	0.347	**0.770**								
	Gustatory	0.379	0.366	0.336	**0.776**							
	Olfactory	0.299	0.376	0.329	0.329	**0.749**						
OMI (structural property)	Autonomy	0.420	0.339	0.268	0.348	0.413	**0.773**					
	Spatial	0.327	0.359	0.416	0.346	0.316	0.325	**0.754**				
	Kinesthetic	0.421	0.413	0.401	0.408	0.429	0.411	0.385	**0.751**			
	Brand experience	0.505	0.530	0.503	0.529	0.513	0.498	0.511	0.551	**0.734**		
	Brand authenticity	0.435	0.373	0.349	0.405	0.413	0.329	0.334	0.375	0.518	**0.773**	
	Social presence	0.357	0.345	0.300	0.379	0.309	0.358	0.297	0.339	0.401	0.401	**0.788**

Note: The AVE square root value is presented in bold on the diagonal of the matrix. SP: social presence; BE: brand experience; BA: brand authenticity.

#### 4.5.3. Model fit.

The indicators in Table 7 show that the model fit was good. The values of CMIN/DF, GFI, AGFI, and RMSEA in the absolute fit index all met the criterion of excellence, indicating that the model fit was superior. The NFI, IFI, TLI, and CFI values in the comparative fitting indicators also exceeded 0.8, indicating a superior value-added fitting of the model. The values of PNFI and PCFI in the parsimonious fitting indexes also reached the standard of excellence. Therefore, the model exhibited a significant level of conformity, indicating a strong alignment between the model and the data [220].

**Table 7:** Model fit.

Index	Expected Value	Actual Value	Fitting Results
Absolute fit index			
CMIN/DF	<3	1.531	Acceptable
GFI	>0.8	0.887	Acceptable
AGFI	>0.8	0.869	Acceptable
RMSEA	<0.08	0.035	Acceptable
Comparative fitting indicators			
NFI	>0.8	0.879	Acceptable
IFI	>0.8	0.955	Acceptable
TLI	>0.8	0.949	Acceptable
CFI	>0.8	0.954	Acceptable
Parsimonious fitting index			
PNFI	>0.5	0.794	Acceptable
PCFI	>0.5	0.861	Acceptable

#### 4.5.4. Pearson correlation.

In this study, the relationships between visual, auditory, tactile, gustatory, olfactory, autonomy, spatial, kinesthetic, and the brand experience were first analyzed in terms of correlations, and the strengths of the correlations between them were expressed through Pearson’s correlation coefficient. According to the analysis results (Table 8), all eight items showed significant relationships with brand experience, with correlation coefficient values between 0.498 and 0.551. Secondly, this study analyzed the correlation between brand experience and brand authenticity. There was a positive correlation between the two items, with a correlation coefficient of 0.518.

**Table 8:** Pearson’s correlation analysis.

	Vis-	Aud-	Tac-	Gus-	Olf-	Aut-	Spa-	Kin-	SP	BE	BA
Visual	1										
Auditory	0.387 **	1									
Tactile	0.316 **	0.347 **	1								
Gustatory	0.379 **	0.366 **	0.336 **	1							
Olfactory	0.299 **	0.376 **	0.329 **	0.329 **	1						
Autonomy	0.420 **	0.339 **	0.268 **	0.348 **	0.413 **	1					
Spatial	0.327 **	0.359 **	0.416 **	0.346 **	0.316 **	0.325 **	1				
Kinesthetic	0.421 **	0.413 **	0.401 **	0.408 **	0.429 **	0.411 **	0.385 **	1			
Social presence	0.357 **	0.345 **	0.300 **	0.379 **	0.309 **	0.358 **	0.297 **	0.339 **	1		
Brand experience	0.505 **	0.530 **	0.503 **	0.529 **	0.513 **	0.498 **	0.511 **	0.551 **	0.401 **	1	
Brand authenticity	0.435 **	0.373 **	0.349 **	0.405 **	0.413 **	0.329 **	0.334 **	0.375 **	0.401 **	0.518 **	1

**p < 0.01. SP: social presence; BE: brand experience; BA: brand authenticity.

### 4.6. Structural equation modeling

**Fig 2:**
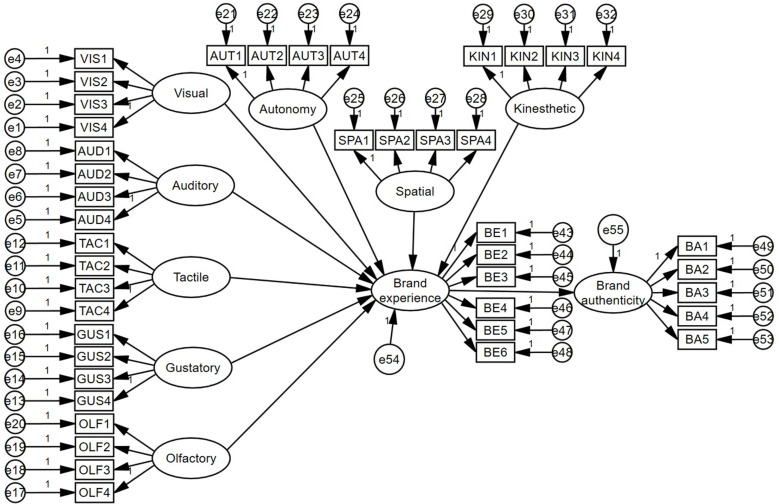
Structural model.

This study described the causal relationship between variables by developing a structural model (Fig 2). The following table displays the model fitting parameters derived from the analysis of the collected questionnaire data using the robust maximum likelihood estimation technique implemented in AMOS 23.0 software (Table 9). The displayed values of most of these fitted parameters met the standard requirements. However, GFI, AGFI, and NFI did not meet the judgment criteria; therefore, the model needed to be corrected to improve its overall fit. Correction was achieved by adding two-by-two correlations between the independent variables, and the corrected model fit parameters are shown below (Table 10). Most of the parameters in the table show values that meet the standard requirements, which indicates that the structural equation model in this paper was a good fit for the sample data obtained from the questionnaire [221].

**Table 9:** Model fit before correction.

Index	Expected Value	Actual Value	Fitting Results
Absolute fit index			
CMIN/DF	<3	2.405	Acceptable
GFI	>0.8	0.75	Reject
AGFI	>0.8	0.722	Reject
RMSEA	<0.08	0.057	Acceptable
Comparative fitting indicators			
NFI	>0.8	0.802	Reject
IFI	>0.8	0.874	Acceptable
TLI	>0.8	0.865	Acceptable
CFI	>0.8	0.873	Acceptable
Parsimonious fitting index			
PNFI	>0.5	0.756	Acceptable
PCFI	>0.5	0.823	Acceptable

**Table 10:** Modified model fit (two-by-two pull correlation of independent variables).

Index	Expected Value	Actual Value	Fitting Results
Absolute fit index			
CMIN/DF	<3	1.55	Acceptable
GFI	>0.8	0.884	Acceptable
AGFI	>0.8	0.867	Acceptable
RMSEA	<0.08	0.036	Acceptable
Comparative fitting indicators			
NFI	>0.8	0.877	Acceptable
IFI	>0.8	0.952	Acceptable
TLI	>0.8	0.947	Acceptable
CFI	>0.8	0.952	Acceptable
Parsimonious fitting index			
PNFI	>0.5	0.799	Acceptable
PCFI	>0.5	0.868	Acceptable

From the results of the structural model (Table 11), the eight paths of “visual, auditory, tactile, gustatory, olfactory, autonomy, spatial, kinesthetic→brand experience” reached significance (*p* < 0.05) with a positive effect. Therefore, hypotheses H1–H8 were verified. In addition, brand experience (β = 0.598, *p* < 0.001) positively affected brand authenticity. Therefore, hypothesis H9 was tested.

**Table 11:** Structural modeling results.

Hypothesized Path	β	B	S.E.	C.R.	*p*
H1. Visual→Brand experience	0.145	0.121	0.039	3.1	0.002
H2. Auditory→Brand experience	0.159	0.139	0.04	3.446	***
H3. Tactile→Brand experience	0.158	0.133	0.038	3.496	***
H4. Gustatory→Brand experience	0.192	0.119	0.028	4.251	***
H5. Olfactory→Brand experience	0.175	0.157	0.043	3.67	***
H6. Autonomy→Brand experience	0.121	0.106	0.041	2.598	0.009
H7. Spatial→Brand experience	0.165	0.112	0.031	3.573	***
H8. Kinesthetic→Brand experience	0.115	0.079	0.036	2.178	0.029
H9. Brand experience→Brand authenticity	0.598	0.713	0.072	9.961	***

***p < 0.001.

### 4.7. Moderating role of social presence

This study aimed to investigate the effect of brand experience on brand authenticity under the influence of multiple factors, considering the moderating role of social presence. The following table outlines the hierarchical regression analysis (Table 12), comprising three models. In Model 1, the focus was solely on the impact of brand experience on brand authenticity. Model 2 introduced social presence as a moderating variable based on Model 1. Model 3 further considered the interaction between brand experience and social presence based on Model 2.

**Table 12:** Hierarchical regression analyses.

	Model 1	Model 2	Model 3
Constant	1.717 ** (7.624)	1.147 ** (4.698)	1.069 ** (4.469)
Brand experience	0.614 ** (12.491)	0.505 ** (9.690)	0.460 ** (8.880)
Social presence		0.230 ** (5.241)	0.265 ** (6.068)
Brand experience × social presence			0.295 ** (4.573)
n	428	428	428
R^2^	0.268	0.313	0.345
Adj. R^2^	0.266	0.309	0.340
F	F (1, 426) = 156.031, *p* = 0.000	F (2, 425) = 96.594, *p* = 0.000	F (3, 424) = 74.385, *p* = 0.000
∆R^2^	0.268	0.044	0.032
∆F	F (1, 426) = 156.031, *p* = 0.000	F (1, 425) = 27.464, *p* = 0.000	F (1, 424) = 20.913, *p* = 0.000

Dependent variable: brand authenticity.

**p < 0.01 t statistics in parentheses.

In Model 1, the independent variable (brand experience) had a significant positive effect (β = 0.614, p = 0.000 < 0.05) on the dependent variable (brand authenticity), which suggests that brand experience had a significant effect on brand authenticity without considering the moderator variable (social presence).

**Fig 3:**
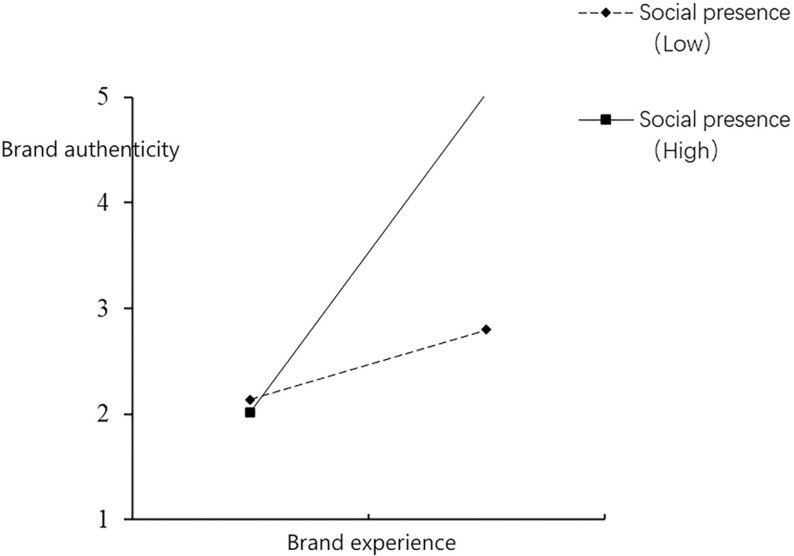
Simple slope diagram.

In Model 3, the interaction term between brand experience and social presence had a significant positive effect on brand authenticity (β = 0.295, *p* = 0.000 < 0.05). When social presence was considered, it was shown to have a moderating effect on this relationship. The magnitude of the effect of brand experience on brand authenticity was significantly greater when social presence was at a high level. In contrast, the magnitude of the effect of brand experience on brand authenticity was relatively small when social presence was low (Fig 3).

## 5. Discussion and implications

### 5.1. Discussion

This study empirically analyzed the conceptual hypothesis model, which revealed how brand experience could be enhanced by and influence brand authenticity. In addition, this study tested the moderating effect of social presence on the relationship between brand experience and brand authenticity, which facilitates the advancement of consumer brand experience. The results of testing the hypotheses of this study are shown below (Table 13).

**Table 13:** Results of hypothesis testing.

	Hypothesis	Result
OMI (sensory property)→Brand experience	H1. Visual mental imagery has a significant positive impact on brand experience.	Accepted
	H2. Auditory mental imagery has a significant positive impact on brand experience.	Accepted
	H3. Tactile mental imagery has a significant positive impact on brand experience.	Accepted
	H4. Gustatory mental imagery has a significant positive impact on brand experience.	Accepted
	H5. Olfactory mental imagery has a significant positive impact on brand experience.	Accepted
OMI (structural property)→Brand experience	H6. Autonomy mental imagery has a significant positive impact on brand experience.	Accepted
	H7. Spatial mental imagery has a significant positive impact on brand experience.	Accepted
	H8. Kinesthetic mental imagery has a significant positive impact on brand experience.	Accepted
Brand experience→Brand authenticity	H9. Brand experience has a significant positive impact on brand authenticity.	Accepted
Moderating role of social presence	H10. Social presence plays a moderating role between brand experience and brand authenticity.	Accepted

The findings suggest that all five reflective components (visual, auditory, tactile, gustatory, and olfactory) of OMI sensory attributes positively influenced brand experience (p<0.05), i.e., Hypotheses H1–H5 were valid. The positive impact of sensory attributes on brand experience could be explained from several perspectives. First, brands can directly elicit an emotional experience from consumers by stimulating these senses,. This emotional response deepens their experience with the brand and created a more profound brand memory. Second, multi-sensory experiences helped to increase engagement, increasing consumers’ investment in brand interactions and their interest in and intimacy with the brand. Finally, brands can create a unified image and increase overall awareness and identity by providing a consistent experience across the different senses. This sensory consistency makes it easier for consumers to form a consistent impression of the brand, deepening their trust and loyalty. These results are consistent with previous research findings. Sensory attributes, as part of mental imagery, are often used as a strategy in brand marketing and the experience economy and have the potential to influence consumer judgment and behavior [10]. The sensory components of the customer experience lie at the heart of brand competitiveness [222]. Elder and Krishna [62] demonstrated that vivid mental imagery fosters deeper emotional connections during brand interactions, enhancing the enjoyment of the brand experience and reinforcing consumers’ identification with and loyalty to the brand. A growing body of research findings have confirmed that modern consumers are focused on the experience generated by purchasing and consuming commercial branded products (goods and services) [223]. Brakus et al. [18] emphasized the unique role of sensory attributes in brand experience. Shahid et al. [105] confirmed that active sensory marketing positively impacts brand experience. The visual, auditory, tactile, gustatory, and olfactory senses strongly influence the perceptions and behavior of the experiencer [224]. Hultén [225] also confirmed that consumers experience brands and services through visual, auditory, tactile, gustatory, and olfactory senses and that sensory cues significantly stimulate consumer approach and behavior. While brand experience is viewed as a multidimensional response to brand-related stimuli, sensory brand experience is unidimensional, emphasizing responses to perceptual attributes rather than dimensions. While sensory experience is a subjective response to various sensory stimuli in the external environment, sensory brand experience focuses on specific responses to brand-related stimuli in the brand environment [46]. In addition, the sensory aspect of customer experience is at the heart of brand competitiveness, and enhancing sensory experience is essential for successful brand management and research [46,222]. These sensory attributes directly influence the customer’s brand experience through different perceptual pathways. For example, high-end retail stores can combine visual aesthetics, background music, fragrances, pleasant tactile materials, and tasting events to create a holistic brand experience that enhances customers’ emotional connection and loyalty. This cross-modal influence between sensory imagery experiences is a crucial area for future research [10].

All three reflective components (autonomy, spatial, and kinesthetic) of the structural attributes in OMI had a positive influence on brand experience (p<0.05), i.e., Hypotheses H6–H8 were valid. First, this study found that autonomy was one of the critical factors in consumers’ participation in brand experience, enhancing their brand engagement and making them feel like active decision-makers rather than passive recipients in the brand experience process. This active engagement enhances the depth of the brand experience and stimulates a stronger emotional connection to the brand. This finding was consistent with previous research. Deci and Ryan’s Self-Determination Theory [136] confirmed that individuals experience higher intrinsic motivation and pleasure when they feel that they have autonomy in their choices. This suggests that when consumers feel that personal values and brand fit are more vital when they have more choices, which enhances their identification and emotional connection with the brand. Gilal et al. [142] emphasized the importance of satisfying consumers’ need for autonomy, and brands that achieved this inevitably enhanced customers’ attachment and advocacy and triggered more positive affective responses. Satar et al. [137] demonstrated that consumers’ degree of autonomy in a brand experience is closely related to their perception of and engagement with the brand. Brands bolster consumer brand experience and emotional engagement by providing personalized products, services, and brand activities that endorse autonomy in choice, promoting a deep emotional connection between the brand and its customer base. Such initiatives further strengthen consumers’ perception and favorability of the brand, thereby increasing the brand’s competitive advantage.

Second, as one of the structural attributes of mental imagery, the spatial construct involves the layout and design of physical and virtual spaces in the brand experience. Reasonable spatial design can guide consumers’ attention and enhance their pleasure in the brand contact process. When customers perceive unique spatial elements and layouts, it heightens their sense of presence and brand recognition, providing a richer and more engaging customer experience. This, in turn, strengthens their memory of and positive feelings toward the brand. This finding is consistent with previous research. Donovan et al. [147] defined the retail environment as an emotional–psychological space, emphasizing the environment’s influence on consumer intentions and consumption behavior. Moreover, a brand’s spatial environment is a dynamic and ever-changing system that is continually being reshaped and redefined by the (intentional or unintentional) intervention of different actors, thus influencing and qualifying the outcome of the brand experience [46]. Zha et al. [46] proposed that the spatial design and interiors of shopping malls are carefully curated to stimulate spatial associations with specific places in personal or collective memory. When consumers are exposed to spatial arrangements, colors, decorations, and overall context, they reinterpreted and give meaning to their aesthetic experience [150]. One study [153] showed that using spatial augmented reality in online stores enhances customers’ immersion and sense of presence, thus enhancing their brand experience. Customers spatially reconstruct their actual perceptions of previous brand experiences, regardless of the shopping method [22]. Finally, kinesthetic attributes, as sensory channels associated with motion in mental imagery, can stimulate consumers’ bodily perceptions and enhance their emotional connection and cognitive experience of the brand. In brand scenarios that involve interaction or experience, kinesthetic experiences create a stronger connection between the consumer and the brand through physical participation, enhancing consumers’ engagement with and emotional attachment to the brand. The results of this study are consistent with previous research. Zeng et al. [155] demonstrated that dynamic brand activities and interactive physical experiences could enhance consumers’ engagement and emotional experience, which deepened their perceptions and favorability towards the brand. Consumers can gain a deeper understanding of a product’s features through physical actions, such as touching, testing, or handling it, allowing them to perceive its authenticity. This leads to more positive emotional responses and an enhanced brand experience [156]. Moreover, Huang and Chung [159] confirmed from the perspective of online shopping that the kinesthetic experience is a critical factor driving pleasure in the virtual shopping experience, which effectively enhances the pleasure of the brand experience by stimulating the three mental states of online consumers’ body schema. For example, in interactive advertising or experiential marketing, active participation and physical interaction makes it easier for consumers to perceive a brand as authentic and reliable. Therefore, by designing innovative and exciting interactive experiences, brands can successfully attract consumers’ attention and established positive brand images and emotional connections. In summary, autonomy, spatial, and kinesthetic, as structural attributes of OMI, all positively influence brand experience to varying degrees.

However, regarding the structural attributes of the OMI scale, only Zhu and Chung [38] used the structural attributes of the OMI scale from the perspectives of mall loyalty and shopper experience, and their study confirmed that only the spatial attribute is applicable. In contrast, the structural autonomy and kinesthetic attributes were not applicable. The current study validated the applicability of the OMI scale from the brand experience perspective, and the results showed that the scale was fully applicable. According to Khalilzadeh et al. [22], the applicability of the scale depends on the imagined subject, environmental factors, and the imaginer’s expectations. Moreover, structural equation modeling can be understood as an extended form of simple regression where multiple paths interact. Even with the same raw data, different combinations of variables could impact the findings. Therefore, subsequent studies and common method bias analyses should be employed to decide whether to include the structure in the scale depending on the situation, and cross-utilization across disciplines would allow the scaled to be further improved and refined.

This study found that customers’ brand experience positively affected brand authenticity (β=0.598, p<0.001), i.e., Hypothesis H9 was valid. The findings suggest that the effective integration of multisensory and structural attributes enriches consumers’ perceptual experience and enhances brand credibility and authenticity. This multisensory experience helps consumers form a comprehensive brand impression, reducing the risk of the brand being perceived as “one-dimensional” or engaging in “false advertising.” Specifically, when consumers perceive consistency across multiple sensory dimensions, they are more likely to view the brand as trustworthy and authentic. Robbins et al. [172] also confirmed that consistency across brand experience dimensions contributes to establishing brand authenticity, as authenticity is a crucial outcome of brand experience that drives success and sustainability. Shamim and Butt [163] noted that customer interactions with the brand’s marketing mix are a crucial part of the brand experience, and the quality of these interactions influences customers’ evaluation of the brand’s credibility. By experiencing the product or service offered, customers can established an actual perception and deep understanding of the brand. Positive brand experiences worked together emotionally, cognitively, and contextually to create an authentic and consistent brand image. This consistent experience reinforced the customer’s belief in the brand’s authenticity because their actual feelings align with the values and promises communicated by the brand. When a distinctive brand consistently delivers on its promises (i.e., demonstrates brand authenticity), consumers tend to have a positive brand experience [166]. Thus, customers’ brand experience is not only about sensory enjoyment but is also directly oriented towards positive perceptions of brand authenticity, building a solid foundation of brand trust. These findings are consistent with those presented in previous studies [14,27]. Safeer et al. [164] confirmed the view of customer brand experience as a driver of brand authenticity as it directly leads to a more positive perception of the brand. When customers feel pleasure and satisfaction while using a brand’s platform, they develop positive feelings, attitudes, and perceptions toward it. These attributes stem from customers’ internal experiences when interacting with the brand [162]. Park et al. [14] also confirmed that a positive brand experience would significantly influence brand authenticity, as this is largely determined by consumers’ actual experiences [171]. Brand authenticity is becoming increasingly important as the need for brands to be digitized and networked increases, as these online platforms are often the primary medium through which consumers interact with brands. If the content and information presented on these platforms is not authentic, consumers might quickly lose interest and trust. Negative feedback can spread like a virus [1]. Therefore, when designing brand experiences, companies should focus on multidimensional mental imagery and emotional connections, ensuring consistent brand messaging across all touchpoints. This approach strengthens consumer trust in the brand’s authenticity, enhancing its market competitiveness and customer loyalty. When consumers engage with an authentic brand and enjoy positive experiences, they develop emotional attachment and affection toward it [27].

In addition, social presence moderated brand experience and authenticity (β=0.295, p=0.000<0.05), i.e., hypothesis H10 was valid. Specifically, when consumers perceive a stronger social presence, the impact of brand experience on brand authenticity becomes more pronounced. This may be because creating a social context enhanced the sense of community and interactivity of the brand experience, which in turn affected customers’ perceptions of brand authenticity. When the brand’s social presence was strong, customers were more inclined to trust the brand and perceive their experience as authentic and credible. Social presence strengthened the influence of the brand’s message in the social network by strengthening the emotional bond between the individual and the brand, which moderated the shaping effect of brand experience on brand authenticity. Thus, social presence enhanced customers’ perceptions of brand authenticity through its mediating effect while creating a profound brand experience. This is consistent with Park et al.’s findings [14], which confirmed that increased social presence strengthens the relationship between brand experience and brand authenticity and that social presence also positively influences behavioral intentions [226] and customer trust in the brand [189]. Social context and other customers influence a customer’s brand experience as customers are more inclined to trust the service or product when feedback from others is positive and similar behaviors from peers are more persuasive [189]. Social presence narrows the perceived gap between customers. It brings a degree of closeness [197] that can make people feel in touch with or understanding of another person’s intentions, perceptions, or emotional states [198]. De Regt et al. [200] also confirmed that customers are more emotionally engaged in the brand experience when they perceive social presence. They are more inclined to foster social interactions, perceived support, and trust [191], perceiving the brand’s authenticity through the establishment of trust [193]. As a result of interpersonal interactions, social presence contributes to a higher level of intimacy, which allows consumers to communicate immediacy or non-immediacy through non-verbal (physical proximity, pictures, and facial expressions) and verbal [201]. This helps companies and researchers to gain a deeper understanding of customers’ behavioral patterns [14]. Therefore, when designing brand experiences, incorporating elements of social interaction or creating a more robust community atmosphere can effectively enhance consumers’ perceptions of brand authenticity. For example, the experience of social presence can be enhanced by inserting descriptions, images, and comments from other customers in social environments where people discuss products and services or by using rich social text. In addition, brands can incorporate short videos, emoticons, and animations to provide more ways for users to shape others’ perceptions of their social presence, further enriching the interactive experience and creating more possibilities [183].

### 5.2. Theoretical implications

This study makes several contributions to the existing literature.

First, regarding the integrated research contribution of multimodal mental imagery, this study fills the gap between multimodal mental imagery and brand experience and authenticity. It bridges the theoretical gap of the OMI scale in the field of brand experience. Most existing research on brand experience focuses on the influence of a single sense or a few senses. In contrast, this study systematically integrates the multi-sensory and structural attributes of OMI for the first time. It proposes a more comprehensive theoretical framework based on OMI, brand experience, and brand authenticity. This study reveals how multimodal mental imagery influences consumers’ perceptions of brand authenticity through multilevel perceptual experiences and validates the applicability and validity of the OMI scale in this field. The findings show that multimodal mental imagery not only enhances the overall perception of a brand but also significantly strengthens the perception of brand authenticity, providing a new perspective on brand management theories and demonstrating the unique appeal of the OMI scale published by Khalilzadeh et al. [22]. This comprehensive perspective fills the gap in previous research analyzing the integration of sensory and structural attributes and deepens the understanding of the complexity of brand experience. Future research should dig deeper into the subtle influences of different perceptual modalities and explore how specific perceptual and structural attributes uniquely influence the brand experience, leading to a more refined understanding of the aspects of mental imagery that impact consumer perception.

Second, in expanding the theoretical boundaries of brand experience and brand authenticity, this study focuses not only on the role of traditional sensory experience but also introduces the dimensions of structural attributes (autonomy, spatial, and kinesthetic) and explores their crucial role in brand experience. It was found that structural attributes play an essential role in enhancing brand authenticity and consistency, especially in helping brands establish a clear identity and long-term brand recognition. This finding extends the boundaries of existing theories of brand experience and brand authenticity beyond sensory-level stimuli to encompass deeper cognitive and structural factors. In addition, this study also broadens the research methodology and theoretical framework of brand experience, provides richer information and empirical support to the existing literature related to brand experience and brand authenticity, and deepens the understanding of brand experience from the perspective of mental imagery, a finding that provides an essential theoretical reference for the study of brand experience and brand authenticity. Therefore, future academic development should explore the interactions between mental imagery and the various dimensions of brand experience. This could involve investigating how individual differences in mental imagery processing affect brand perceptions and preferences. Integrating theoretical frameworks that span across the fields of cognitive psychology and neuroscience could deepen our understanding of the mechanisms behind sensory experiences in brand environments.

Third, this study provides a new perspective for branding in the digital era. The moderating role of social presence provides a new perspective on branding in the digital era, emphasizing the importance of online social environments for brand experience and authenticity. Moreover, social presence can foster positive attitudes and purchase intentions towards products and brands [14]. We deepen our understanding of brand experience and authenticity through new empirical evidence and findings. These findings provide new research methods and theoretical perspectives for an in-depth study of the relationship between brand experience and consumer behavioral intentions, as well as specific theoretical guidance for brand managers and business executives to assist them in better understanding brand consumption and customer behavior patterns.

Fourth, this study identifies a profound impact on brand management practice. The theoretical framework of this study provides brand managers with new strategic tools and theoretical support, not only to enhance the attractiveness of the brand by optimizing the sensory experience but also to improve the authenticity and consistency of the brand by adjusting the brand structural attributes (e.g., the way of brand storytelling, the layout of the brand elements, and the tempo of the brand communication). This multi-dimensional management strategy helps brands build solid brand equity in a complex and changing market environment.

Finally, this study lays a comprehensive theoretical foundation for future research: This study also lays a foundation for future research on how sensory and structural attributes affect brand experience in different cultural contexts and market environments outside of China. By revealing the unique role of structural attributes in brand authenticity, this study provides a theoretical basis for further research on how these attributes can be applied universally across cultural differences in global markets. This comprehensive theoretical framework enriches academic research on brand management and provides rich directions for future empirical studies.

### 5.3. Practical implications

This study provides practical management insights and practical guidance for brand managers and marketing practitioners in the areas of brand management and experiential marketing, with important practical implications.

First, regarding brand experience optimization, research findings show that OMI can significantly enhance brand experience and brand authenticity. Therefore, brand managers and marketers can draw on this finding to enhance customer experience by improving brand design and operations. Regarding mental imagery, in particular, brands can track the significant impact of their marketing and service efforts in more detail by creating pleasurable and memorable sensory experiences, optimizing spatial layouts, and enhancing kinesthetic experiences. For example, multi-sensory pathways such as visual design, sound signage, unique materials and branded fragrances, well-designed shopping spaces, and dynamic displays can create a more immersive brand experience. This helps improve overall brand perception and enhances brand authenticity, thereby increasing market competitiveness and maintaining brand sustainability. Although multi-sensory experiences have apparent advantages in theory, effectively integrating these sensory elements in practice is still challenging. Brand managers must fully consider the synergy between different sensory elements to ensure proper integration, which may result in a cohesive experience or a sensory burden for consumers.

Second, the experience should be customized according to specific sensory elements: The sensory attributes of mental imagery may have different impacts in different markets and cultural contexts. Therefore, brand managers should consider implementing customized sensory strategies based on local consumers’ cultural characteristics and sensory preferences in global marketing. The flexible application of such strategies can better meet consumers’ individual needs, thus improving the brand’s market adaptability and consumer satisfaction. Furthermore, for brand managers, in addition to the company’s efforts (e.g., advertising or promotions), it is crucial to consider the role of the consumer in shaping the brand experience [161]. Therefore, this study suggests focusing on specific sensory elements that influence the brand experience, optimizing management, and adapting marketing strategies from the consumer’s perspective to enhance these elements, whether they are visual, auditory, tactile, gustatory, or olfactory, to create a more immersive and memorable brand experience to meet customers’ psychological expectations. This may involve engaging consumers through innovative technologies such as virtual or augmented reality, which amplify the impact of sensory elements. This helps create an innovative and forward-looking image of the brand but also helps to track the impact of service and marketing performance in more detail [22].

Third, social presence for authentic branding should be utilized. This study also suggests that social presence is important in moderating the relationship between brand experience and authenticity; this provides new practical guidance for managers and marketers to utilize social presence to enhance brand authenticity. In the digital and social media environment, engaging customers through text, images, customer reviews, and shared testimonials; promoting user-generated content; and cultivating a sense of community can boost social presence and boost the brand’s overall image, perceived authenticity, and market competitiveness. These efforts also help maintain the emotional bond between consumers and the brand, thereby contributing to increased consumer loyalty.

Fourth, a global strategy should be culturally sensitive. Brand managers should recognize the importance of cultural sensitivity given that the study data were only collected within China. When implementing strategies based on sensory and structural elements, it is crucial to consider cultural variations in how people perceive and react to mental imagery stimuli. This requires customizing strategies to fit the cultural context of different markets.

Finally, strategies should be continuously monitored and adjusted. Given the dynamic nature of consumer preferences and technological advances, brand managers should adopt a constant monitoring and adjustment approach. Regularly evaluating the effectiveness of sensory and structural elements and social presence in shaping brand experience can help ensure that marketing strategies can be adjusted promptly. This adaptive approach ensures that brands remain relevant despite changing consumer expectations. At the same time, it is important to continually adjust the balance between investment and return in resource allocation and technological implementation to ensure the sustainability and effectiveness of sensory strategies.

### 5.4. Limitations

Despite this study’s theoretical contributions and practical implications, as with most research, there were limitations.

First, the data and models in this study were collected and validated in China, and the limitation of the sample scope may limit the generality and universality of the findings. Future studies should select countries and regions with different cultural backgrounds for data collection and model validation to enhance the scientific validity of the findings.

Second, due to the questionnaire used in this study and the uniqueness of the OMI scale, the data collected may be influenced by the mental imagery of the subjects. In terms of research methodology, future studies can triangulate data by combining qualitative research methods, such as in-depth interviews, to increase the analysis’s breadth and depth. In terms of data collection related to mental imagery, psychological simulations can be conducted through virtual reality (VR) and augmented reality (AR) technologies, and methods such as eye tracking (ET) and electroencephalogram (EEG) can also be utilized to improve the accuracy of the data and imagery measurements from a psychophysiological perspective.

Finally, as marketing and consumer concepts continue to evolve along with the widespread use of social media and innovations in digital technology platforms, brand experiences across different brand types and different industries have become more novel and varied and continue to attract academic research attention. Future research could broaden the structural aspects of brand experience explored in this study by identifying which specific dimensions are influenced by mental imagery. Additionally, it would be beneficial to establish the logical relationship between variables of OMI within the framework of multidimensional brand experience, encompassing emotional, intellectual, and behavioral components.

## 6. Conclusion

This study is the first to use the OMI scale in the field of brand experience. The findings show that all five sensory attributes of the OMI, namely visual, auditory, tactile, gustatory, and olfactory, positively affect brand experience; in terms of structural attributes, autonomy, spatial, and kinesthetic positively affects brand experience. This not only fills a theoretical gap in the field of brand experience but also provides brand managers with practical guidance on optimizing sensory experience. Brand managers can enhance these perceptual elements through innovative technologies and marketing techniques to create more profound and creative brand experiences, particularly with respect to the visual, auditory, and tactile senses.

The findings of this study emphasize the positive impact of brand experience on brand authenticity. In particular, social presence plays a critical moderating role in this relationship. This allows brand managers to enhance their brand’s perceived authenticity through social interaction and social presence enhancement. Active participation in online social platforms and encouraging user-generated content will help build an authentic and credible brand image and increase consumer loyalty to the brand. This finding provides new insights into brand management practices and a rich theoretical foundation for future research.

In today’s competitive market environment, a brand’s differentiated competitive advantage often depends on consumers’ deep perception and experience of the brand. The results of this study provide brand managers with practical strategic suggestions that optimizing and integrating different mental imagery can achieve significant results in shaping the brand image and enhancing brand authenticity.

Future research could further explore the applicability of OMI in different cultural contexts or delve into the unique contributions of different sensory and structural attributes to brand experience. This will not only help validate the generalizability of the current findings but will also broaden the scope of the application of brand management practices. Second, the research results may face challenges such as integration difficulties and consumer differentiation needs in practical application, so means of effectively incorporating these mental imagery elements into branding strategies still require further exploration and practice. In addition, understanding mental imagery formed through augmented reality and virtual reality technologies will continue to be an exciting area of research as these technologies offer unlimited possibilities for both brands and consumers.

## Supporting information

S1. File Operationalization of measures. (PDF)

S1. Data Survey results. (XLSX)
