# Supplementary material for: Enhancing brand experience and brand authenticity: The role of octomodal mental imagery and social presence
Source: PLoS One. 2025 Apr 29;20(4):e0321883. doi: 10.1371/journal.pone.0321883 (PMC12040128; doi:10.1371/journal.pone.0321883)
Supplement: S1. File — (PDF) [file pone.0321883.s001.pdf]

Appendix 1.

Questionnaire

1. **Basic demographic information.**

Gender：

Male

Female

Age：

18-25 years old

26-35 years old

36-45 years old

Over 45 years old

Marital status：

Single

Married

Education level:

Junior high school or below

High school or junior college;

Junior college

Undergraduate

Master's degree or above

Average monthly income (including allowances, dividends and other forms of income):

5000 and below

5001-10000；

10001-20000；

20001-30000；

30001-50000；

50001 and above

2. Measurement scale.

Constructs	Item<br>(From 1 = Strongly Disagree to 7 = Strongly Agree)	Sources
OMI (Octomodal Mental Imagery)Test: Please close your eyes and imagine that you are searching for, purchasing a branded item, or receiving a corresponding service. Take a moment to create a detailed mental image. When you have finished creating it, open your eyes and very carefully choose the option that best explains your mental scenario and its details.		
	OMI (Sensory Property)	
Visual	1. My mental image is detailed.<br>2. In my mental image, I vividly see everything.<br>3. My mental image is a sharp image.<br>4. In my mental image, most of the elements/things are noticeable.	Adapted from (Khalilzadeh et al., 2023)
Auditory	1. In my mental image, I hear many different sounds/noises.<br>2. In my mental image, the sound(s)/noise(s) I hear is/are very clear.<br>3. In my mental image, the sound(s)/noise(s) I hear is/are intense.<br>4. In my mental image, the sound(s)/noise(s) I hear is/are detailed.	
Tactile	1. In my mental image, I touch elements/things.<br>2. In my mental image, I feel the textures of elements/things.<br>3. In my mental image, I feel many different textures.<br>4. In my mental image, I have a clear understanding of the coarseness of the elements.	
Gustatory	1. In my mental image, the flavor(s) I taste is/are detailed.<br>2. In my mental image, the flavor(s) I taste is/are very intense.<br>3. In my mental image, most of the flavors I taste are very noticeable.<br>4. In my mental image, I taste many different flavors.	
Olfactory	1. In my mental image, smell(s) is/are very real.<br>2. In my mental image, the odor(s)/scent(s) I smell is/are very detailed.<br>3. In my mental image, the odor(s)/scent(s) is/are very clear.<br>4. In my mental image, I smell many different odors/scents.	
	OMI (Structural Property)	
Autonomy	1. In my mental image, I can easily dismantle anything I want.<br>2. In my mental image, I can easily change the sizes of any entity.<br>3. In my mental image, I can easily change the shape of elements/things.<br>4. In my mental image, I can easily rotate elements/things.	Adapted from (Khalilzadeh et al., 2023)
Spatial	1. In my mental image, I have a precise idea of the spatial surroundings of elements/things.	

	2. In my mental image, I have a precise idea of the locations of elements/things.<br>3. In my mental image, I have a precise idea of the directions of elements/things.<br>4. In my mental image, I have a precise idea of the distances of elements/things from each other.	
Kinesthetic	1. In my mental image, everything is alive and animated.<br>2. My mental image is animated and movie-like.<br>3. In my mental image, I clearly see gestures.<br>4. In my mental image, I clearly see postures.	
	Variables	
Brand experience	1. This brand makes a strong impression on my visual and other senses.<br>2. This brand induces feelings and sentiments.<br>3. This brand is an emotional brand.<br>4. I engage in physical actions and behaviors when I use this brand.<br>5. I engage in a lot of thinking when I encounter this brand.<br>6. This brand does not make me think.	Taken from (Brakus et al., 2009)
Brand authenticity	1. The brand possesses a clear philosophy that guides the brand's promise.<br>2. The brand knows exactly what it stands for and does not promise anything which contradicts its essence and character.<br>3. Considering its brand promise, the brand does not pretend to be someone else.<br>4. Considering its brand promise, the brand does not curry favor with its target group; moreover, it shows self-esteem.<br>5. The brand distorts itself to match contemporary market trends.	Taken from (Schallehn et al., 2014)
	Moderator Variable	
Social presence	1. There is a sense of sociability in the seller’s network.<br>2. There are many other buyers sharing information related to the brand.<br>3. Interacting with the seller through social networks allows me to understand the seller’s attitude.<br>4. Interacting with them through social networks, I can imagine what they look like.	Adapted from (Lu et al., 2016)
